# Brain Capillary Networks Across Species: A few Simple Organizational Requirements Are Sufficient to Reproduce Both Structure and Function

**DOI:** 10.3389/fphys.2019.00233

**Published:** 2019-03-26

**Authors:** Amy F. Smith, Vincent Doyeux, Maxime Berg, Myriam Peyrounette, Mohammad Haft-Javaherian, Anne-Edith Larue, John H. Slater, Frédéric Lauwers, Pablo Blinder, Philbert Tsai, David Kleinfeld, Chris B. Schaffer, Nozomi Nishimura, Yohan Davit, Sylvie Lorthois

**Affiliations:** ^1^Institut de Mécanique des Fluides de Toulouse (IMFT), Université de Toulouse, CNRS, Toulouse, France; ^2^Nancy E. and Peter C. Meinig School of Biomedical Engineering, Cornell University, Ithaca, NY, United States; ^3^Department of Biomedical Engineering, University of Delaware, Newark, DE, United States; ^4^Toulouse NeuroImaging Center (TONIC), Université de Toulouse, INSERM, Toulouse, France; ^5^Department of Anatomy, LSR44, Faculty of Medicine Toulouse-Purpan, Toulouse, France; ^6^Department of Neurobiology, George S. Wise Faculty of Life Sciences, Tel-Aviv University, Tel Aviv, Israel; ^7^Department of Physics, University of California, San Diego, La Jolla, CA, United States

**Keywords:** cerebral cortex, capillary network, capillary loop, capillary network model, biomimetic network

## Abstract

Despite the key role of the capillaries in neurovascular function, a thorough characterization of cerebral capillary network properties is currently lacking. Here, we define a range of metrics (geometrical, topological, flow, mass transfer, and robustness) for quantification of structural differences between brain areas, organs, species, or patient populations and, in parallel, digitally generate synthetic networks that replicate the key organizational features of anatomical networks (isotropy, connectedness, space-filling nature, convexity of tissue domains, characteristic size). To reach these objectives, we first construct a database of the defined metrics for healthy capillary networks obtained from imaging of mouse and human brains. Results show that anatomical networks are topologically equivalent between the two species and that geometrical metrics only differ in scaling. Based on these results, we then devise a method which employs constrained Voronoi diagrams to generate 3D model synthetic cerebral capillary networks that are locally randomized but homogeneous at the network-scale. With appropriate choice of scaling, these networks have equivalent properties to the anatomical data, demonstrated by comparison of the defined metrics. The ability to synthetically replicate cerebral capillary networks opens a broad range of applications, ranging from systematic computational studies of structure-function relationships in healthy capillary networks to detailed analysis of pathological structural degeneration, or even to the development of templates for fabrication of 3D biomimetic vascular networks embedded in tissue-engineered constructs.

## 1. Introduction

Archaeologists can understand past human economic and socio-political behavior, or resilience of ancient societies to strong perturbations such as repeated drought, from the organization of their infrastructures such as roadways, water supply or sewage networks (Dillehay and Kolata, 2004). In the same way, the mechanisms of cognition in health and disease might ultimately be informed by studying the brain micro-vascular system.

This system provides a highly integrated and dynamic infrastructure for the distribution of blood: it supplies oxygen, nutrients and, in some cases, drugs to every cell in the brain, and ensures the removal of metabolic waste. Since the brain lacks any substantial energy reserve, the cerebral microcirculation also acts as a short-term regulation system, which responds quickly and locally to the metabolic needs of neurons (Hillman, 2014; Rungta et al., 2018). In imaging neuroscience, changes in blood supply are thus considered as a surrogate for changes in neuronal activity, providing a unique way to observe the functioning brain.

The brain microvascular system is also involved in disease, including stroke and neurodegenerative disease, through vascular damage, such as capillary occlusions and progressive rarefaction (Østergaard et al., 2016; Cruz Hernández et al., 2019), and decrease in regulation efficiency (Farkas and Luiten, 2001; Iadecola, 2004). Together, these act to reduce blood flow and the availability of vital nutrients, which, on one hand, plays a key role in disease progression (Zlokovic, 2011; Cruz Hernández et al., 2019) and, on the other hand, makes it difficult to interpret functional imaging data in patient populations (D'Esposito et al., 2003).

Consistent with its functions of distribution and exchange, the microvascular system includes several architectural components. The arterioles form a quasi-fractal hierarchy of vessels (Nishimura et al., 2007; Blinder et al., 2010; Lorthois and Cassot, 2010; Shih et al., 2015) whose diameter decreases at each successive bifurcation, thus minimizing the time for supplying resources (Lorthois and Cassot, 2010). These vessels feed into the capillary network, a dense, mesh-like, three-dimensional (3D) interconnected structure, which is space-filling above a characteristic length scale of order 25−75μm (Lorthois and Cassot, 2010). This ensures that no point in the tissue is further than half this characteristic length from the nearest vessel, due to the diffusion-limited distance of oxygen transport in oxygen consuming tissue. Their mesh-like structure gives the capillaries, the smallest vessels in the vasculature with a diameter ~5μm, an extremely large surface area facilitating their vital role in nutrient exchange (Popel and Johnson, 2005). De-oxygenated blood then drains into the venules, which broadly mirror the architecture of the arterioles.

These basic principles apply to a large variety of mammals, from rodents to humans, where the main difference in vascular organization described so far is the ratio between arterioles and venules which feed/drain a given region (Hartmann et al., 2017). Beyond these principles, thorough characterization of microvascular structure in the cortex is still incomplete. Thanks to the increasing number of vascular anatomical datasets in the literature (e.g., Cassot et al., 2006; Mayerich et al., 2008; Tsai et al., 2009; Blinder et al., 2013; Xiong et al., 2017; Di Giovanna et al., 2018), the arterioles and venules both within the cortex (Cassot et al., 2010; Hirsch et al., 2012; Lorthois et al., 2014b) and at the level of the pial surface (Blinder et al., 2010) have been rigorously analyzed. However, despite their key role in supplying neurons with the required nutrients, there has been much less focus on the dense, complex mesh of capillaries. Previous studies of 3D cortical capillaries have principally been qualitative (e.g., Duvernoy et al., 1981), or limited to the characterization of global spatial properties, such as their space-filling nature, density, or diameter and length distributions (Lauwers et al., 2008; Lorthois and Cassot, 2010), with few insight on topology. One notable exception (Blinder et al., 2013) studied minimal loops and vessel resistance distributions to conclude that the capillaries form a highly interconnected mesh with no structural correlation to the location of cortical columns.

As a result, current understanding of the architectural organization of healthy brain capillary networks within the cortex is limited to a few striking features:

1. Brain capillary networks are approximately isotropic anastomosing networks whose vertices mainly have three connections (e.g., Duvernoy et al., 1981; Blinder et al., 2013);
2. They are space-filling above a cut-off length of order 25−75μm (e.g., Lorthois and Cassot, 2010);
3. They approximately demarcate convex tissue domains with a characteristic length of similar order (in contrast to tumor networks whose tissue domains are multi-scale in nature, e.g., Baish et al., 2011).

This makes it difficult to build a precise mental representation of these networks that can materialize into a relevant generic capillary network model (or *geometric archetype* in the words of Baish et al., 2011). Besides a better understanding of the fundamental organization of the cortical capillaries, such a generic network model is also needed for fundamental studies focused on understanding how structural differences between brain areas, organs, species or patient populations translate into functional differences with regard to blood flow, blood/tissue exchange, and associated imaging signals, e.g., in BOLD fMRI.

Similarly, implementation of image-guided, biofabrication techniques (Brandenberg and Lutolf, 2016; Heintz et al., 2016, 2017; Pradhan et al., 2017; Hoon et al., 2018) provides the ability to generate 3D, biomimetic vascular networks embedded in tissue-engineered constructs. These microphysiological systems could be useful for investigating the impact of capillary architecture and hemodynamics on complex biological processes in the brain, e.g., transport across the blood brain barrier.

Hence, the goals of this paper are:

1. To thoroughly characterize the structure and function of healthy cerebral capillary networks in both mice and humans, thereby identifying the similarities;
2. To generate synthetic capillary networks with equivalent properties via a generic method which is not tuned to a specific dataset, thereby evidencing key common organizational features among mice and humans.

These goals are inherently inter-linked and must be developed in parallel, to overcome the following challenge. A geometric archetype is necessary to guide definition and scaling of an appropriate set of metrics for characterizing both the structural and functional properties of brain capillary networks. On the other hand, thorough characterization of these properties from real experimental data is needed to ensure the relevance of this geometric archetype.

**Figure 1:**
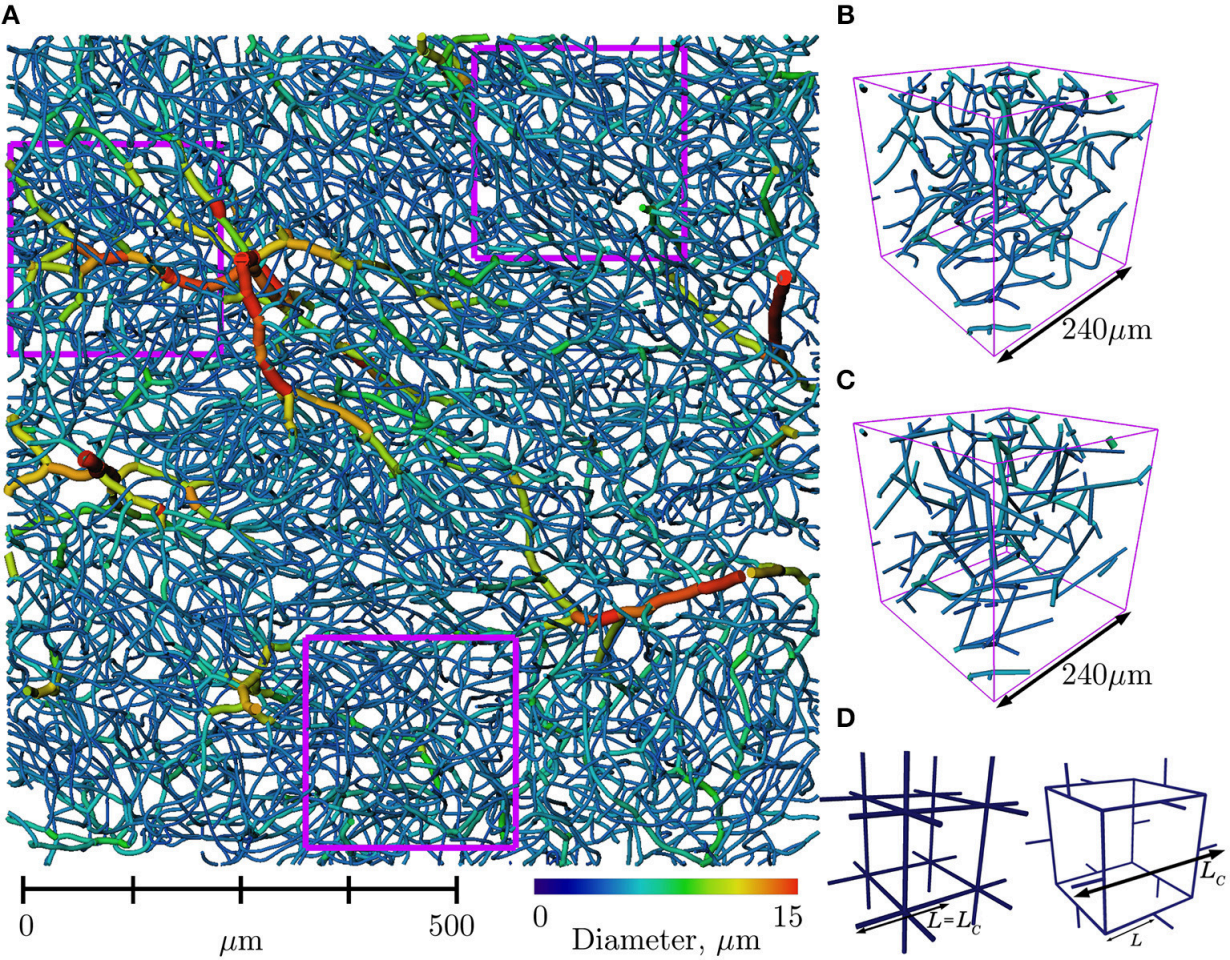
**(A)** Section of mouse cerebral cortex from Tsai et al. (2009), viewed from above the pial surface (upper section of cortex and surface vessels removed for visualization purposes) and with vessels color-coded according to diameter. Three regions of interest (ROIs) of size 240 × 240 × 240μm^3^ are outlined in fuschia. **(B)** One ROI in further detail, with the same color scheme. **(C)** The same ROI with vessels straightened. Tortuosity was ignored in our analysis of network properties. **(D)** Simple, periodic grid-like lattice networks enable analytical derivation of scaling properties (see section 2.2.2): CLN with 2 × 2 × 2 elementary cells (left), and 1 elementary cell of the PLN (right).

**Figure 2:**
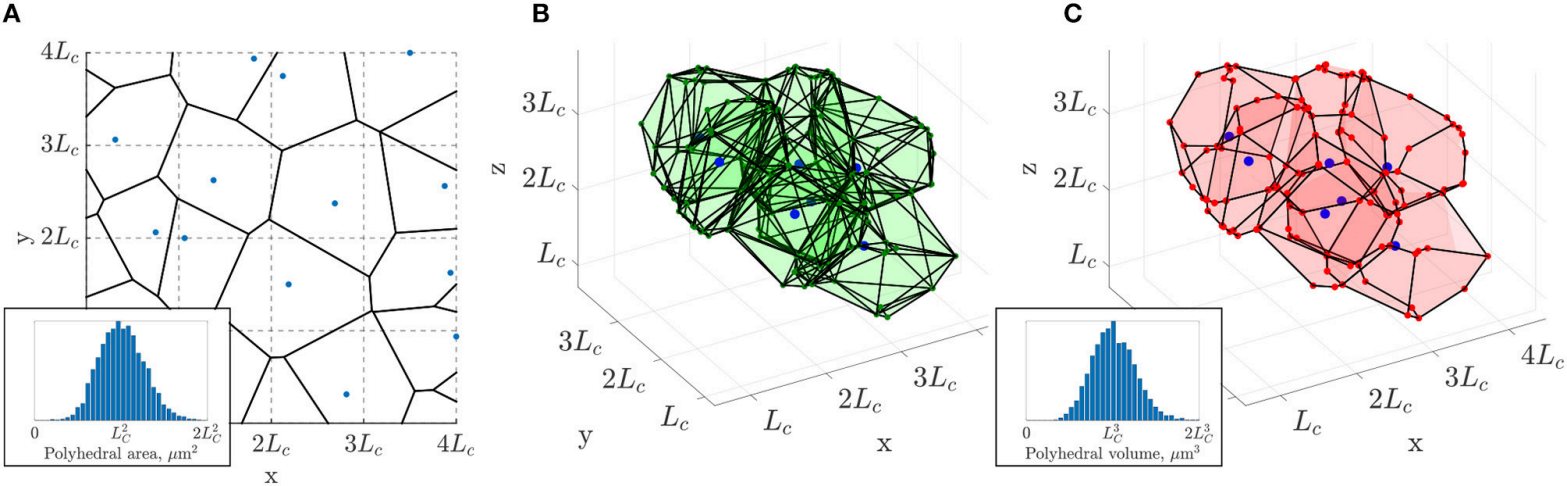
3D extension of the 2D constrained Voronoi method of Lorthois and Cassot (2010). **(A)** Example of a 2D Voronoi diagram (thick black lines) generated from an array of seed points (in blue), one randomly placed in each cell with side length *L*_*C*_ of a square grid (dashed lines). Inset: the distribution of polygonal areas, collected over 80 networks of size ${\left(3.2L_{c}\right)}^{2}$, followed a Gaussian distribution with mean of approximately $L_{C}^{2}$ (4473 polygons in total). **(B)** In 3D, a subset of polyhedra of the Voronoi diagram generated from the seed points in blue, one randomly placed in each cell with side length *L*_*C*_ of a cubic grid (not showing all polyhedra for visualization purposes). **(C)** The same polyhedra with faces merged according to minimum angle and face area criteria as detailed in section 2.2 and section S1 of the Supplementary Material. Inset: the distribution of polyhedral volumes, collected over 10 networks of size ${\left(3.2L_{C}\right)}^{3}$, followed a Gaussian distribution with mean approximately $L_{C}^{3}$ (4, 408 polygons in total).

Therefore, the present paper is organized as follows. First, we describe the anatomical capillary datasets from mice and human cerebral cortex (section 2.1; mouse data shown in Figures 1 A-C). Then, we postulate that the current understanding of their architectural organization, as described by the three general features above, is sufficient to generate model networks replicating not only the morphological and topological properties of cerebral capillary networks, but also their flow and transport properties. Based on this postulate, we introduce in section 2.2, a constrained Voronoi-based method for generating 3D synthetic capillary networks with these three features, as summarized in Figure 2. Simpler, periodic grid-like lattice networks are also introduced (Figure 1D) to enable analytical derivation of metrics and associated scaling properties.

In section 2.3, we present a comprehensive set of quantitative metrics enabling characterization of network structure and function, that is: morphometrical metrics for the tissue (e.g., mean extravascular distances) and the capillary network (e.g., mean vessel length, length density); topological metrics (e.g., number of edges per capillary loop); flow metrics (e.g., velocity, permeability); mass transfer metrics (e.g., intravascular transit times, mass exchange coefficient) and robustness to occlusions (post vs. pre-occlusion flow ratio).

In the Results, these metrics are used in combination to demonstrate that, with appropriate scaling, mice and human capillary networks have similar properties. Moreover, we show that these properties can be matched by synthetic networks, and even to some extent simple lattice networks, demonstrating that only a few organizational requirements are sufficient to fully reproduce the fundamental properties of cerebral capillary networks.

## 2. Materials and Methods

As described above, we first introduce the anatomical datasets used (section 2.1), then present the methods for generation of synthetic and lattice networks (section 2.2), before defining the metrics used to quantify and compare network properties (section 2.3). For clarity, in the latter sections, we focus on the general strategy and highlight the main ingredients. Further details not essential for understanding the present approach are given in section S1 of the Supplementary Material and in Appendix A, respectively. Unless otherwise indicated, the procedures described were implemented in a custom-built C++ code (Peyrounette et al., 2018).

### 2.1. Anatomical Datasets

Firstly, capillary ROIs were manually extracted from mouse and human anatomical datasets as follows.

#### 2.1.1. Mouse Data

Vascular networks from the mouse somatorsensory cortex were previously obtained using a morphological-preserving vascular cast protocol (Tsai et al., 2009; Blinder et al., 2013). Briefly, the animals were euthanized with an overdose of pentobarbital. They were transcardially perfused at a rate of 0.5 ml/s to match the mouse heart output, with warm (37°C) saline until all blood was cleared (~ 40–50 ml) and then with an excess of 20 ml of vascular casting perfusate, previously prepared by conjugating fluorescein-labeled-albumin (no. A9771; Sigma) with a 2% (w/v) solution of porcine gelatin (no. G1890; Sigma). The gel was allowed to solidify for 15 min while the animal was tilted down and immersed in an ice-cold water bath. Next, the head was severed at the level of the neck and moved overnight for fixation in 4% paraformaldehyde (PFA). The following day, the brain was removed from the skull under a fluorescent binocular (Zeiss Discovery 8). In order to preserve the dura and pial vasculature intact, the dissection was conducted guided by the fluoresce signal from the vascular cast which allowed the careful identification of dura to skull attachment places that were crucial to disconnect prior to removal of the corresponding skull bone. Importantly, the bone was removed in small fractions, starting from the dorsal aspect and working in a circular fashion while progressing rostral until the whole brain was exposed. The brain was then moved back to PFA for 24 h. Images of the pial vasculature were obtained to serve as reference for subsequent optical sectioning of thick slabs, using two-photon laser scanning microscopy (TPLSM), at a resolution of 1 μ*m*^3^. After data segmentation and vectorization of the vascular networks as described by Tsai et al. (2009), vessel diameters were corrected to match values observed *in vivo* using an histogram matching approach (Cruz Hernández et al., 2019).

Arterioles and venules within the cortex were differentiated from the capillary mesh by manually classifying surface arteries/veins and then following connecting vessels downstream/upstream while vessel diameter was above a specified minimum threshold (7.2 μm), chosen for this dataset so that the resulting trees did not contain any loops (Cruz Hernández et al., 2019). Seven ROIs were selected from two cortical zones at cortical depths of over 650μm, to avoid vessels classified as arterioles and venules and extract the largest possible sections which only contained capillaries. Nonetheless, ROIs were limited to a size of 240 × 240 × 240μm^3^. The location of three such ROIs are shown in Figure 1A.

#### 2.1.2. Human Data

Human data was obtained from the lateral part of the collateral sulcus (fusiform gyrus) of the temporal lobe as described in Cassot et al. (2006). Briefly, 300 μm-thick sections of a human brain injected with Indian ink, from the collection of Henry Duvernoy (Duvernoy et al., 1981), were imaged by confocal laser microscopy, with a spatial resolution of 1.22 × 1.22 × 3 μm. The brain came from a 60 year old female who died from an abdominal lymphoma with no known vascular or cerebral disease. The procedures used to obtain a complete automatic reconstruction of the vascular network in large volumes (1.6 mm^3^) of cerebral cortex, i.e., mosaic M1 in Cassot et al. (2006) have been described in detail elsewhere (Cassot et al., 2006; Fouard et al., 2006). The mean radius and length of each segment were rescaled by a factor of 1.1 to account for the shrinkage of the anatomical preparation (Lorthois et al., 2011a). The main vascular trunks were identified manually and divided into arterioles and venules according to their morphological features, following Duvernoy's classification (Duvernoy et al., 1981; Reina De La Torre et al., 1998). Arteriolar (resp. venular) trees within the cortex were then differentiated from the capillary mesh as above, with a threshold value of 9.9μm (Lorthois et al., 2011a).

From this classification, the largest possible capillary-only zones were identified, being limited in the *x* and *y* directions by the need to avoid arterioles and venules, and in the *z*-direction by the imaging depth. Since the slice of cortex studied was originally selected for its many large arborescences, this made difficult the extraction of capillary-only zones. Only four ROIs, of size 264 × 264 × 207μm, were identified, one at a cortical depth of 300μm and three at a depth of over 1 mm. These regions were segmented from the raw images using DeepVess (Haft-Javaherian et al., 2019), a 3D deep convolutional neural network architecture for vasculature segmentation. The segmentation was then manually corrected by direct comparison with the raw images in Avizo to ensure that the network connectivity was well-reproduced. Despite this, the final segmentation was inevitably less reliable for vessels near the limit of the confocal imaging depth due to the associated attenuation.

### 2.2. Synthetic Capillary Networks

As summarized in the Introduction, we hypothesize that the minimal organizational requirements of healthy cerebral capillary networks are that they are isotropic, three-connected and space-filling with approximately convex extravascular domains. The physiological hypothesis is that this ensures that no point in the oxygen consuming tissue is further than the diffusion-limited distance of oxygen transport from the nearest vessel.

To generate such networks, a method was sought to derive a tessellation of space into semi-regular “supply regions,” where capillaries lie along the boundaries separating these regions. Voronoi diagrams provide a simple way to achieve this, as illustrated in Vrettos et al., 1989; Kou and Tan, 2010; Wu et al., 2012, and have been previously employed to generate 2D capillary networks (Lorthois and Cassot, 2010). We first present this method and its generalization to 3D, before defining grid-like lattice networks whose properties can be studied analytically. All these networks are defined up to a constant factor, the characteristic length *L*_*C*_, which only controls the network scaling, and has no impact on topology. The exact choice of *L*_*C*_ is non-trivial and will thus be investigated in the Results.

#### 2.2.1. Generation of Synthetic Capillary Networks Using Voronoi Diagrams

A Voronoi diagram or tessellation is the unique graph partitioning the space into polyedra based on distance to pre-selected “seed” points so that each polyhedra associated to a given seed is the region consisting of all points closer to that seed than to any other (Okabe et al., 2008). Here, the edges of the resulting Voronoi polyhedra (or polygons in 2D) represent the capillaries.

##### 2.2.1.1. 2D case

The constrained Voronoi-based approach of Lorthois and Cassot (2010) consists of the construction of a 2D Voronoi diagram from a set of uniformly distributed seed points under the strong constraint that there is only one point in each cell of size $L_{C}^{2}$ in a square grid (Figure 2A). The characteristic length *L*_*C*_, which controls the network scaling, corresponds roughly to twice the typical maximum inter-capillary distance. From Lorthois and Cassot (2010), it is understood to be at least equal to the mean capillary length and broadly in the range 50−100μm.

The constrained spacing of initial seed points yields an isotropic, homogeneous and space-filling network, which results in a Gaussian distribution of Voronoi polygon areas with mean approximately $L_{C}^{2}$ (Figure 2A, inset). In contrast, tumorous microvascular networks, which are not space-filling, display a non-Gaussian distribution of extravascular spaces with some very large gaps in the network, inhibiting tractable drug delivery to the tissue (Baish et al., 2011).

The resulting 2D networks are also quasi-regular in the sense that almost all junctions are bifurcations i.e., have three-connectivity. The network structure is randomized but sufficiently ordered that the networks are vectorizable (Moukarzel and Herrmann, 1992), i.e., topologically equivalent to a strongly perturbed square grid (Schaller and Meyer-Hermann, 2004), and homogeneous at the network scale. In short, the resulting networks possess all the desired features, except for being two-dimensional.

##### 2.2.1.2. Extension to 3D

This method can be generalized to 3D by dividing a 3D region into a regular grid comprising sub-cubes with edges of length *L*_*C*_ (section S1.1. in the Supplementary Material). The resulting 3D Voronoi tessellation fulfills all the desired properties (isotropic, space-filling, convex extravascular domains), but has high connectivity. Many vertices have up to 5 connections (Figure 2B), in contrast to cerebral capillary networks. Additionally the networks contain many unrealistic features, such as closely-located vertices, short edges, sharp branching angles and high vascular density. In brief, these networks are overly-precise tessellations of space with the associated polyhedra strictly defining convex monodisperse extravascular volumes (Figure 2B).

Our hypothesis is that sub-networks with mostly three-connectivity can be extracted from these initial networks while retaining the desired characteristics (Figure 2C). For that purpose, edge and vertices were randomly merged, pruned or added under geometrical constraints as described below, so that the final 3D network retains tissue volumes with a Gaussian distribution that scales with $L_{C}^{3}$, and also achieves three connectivity (Figure 2C). This procedure was developed in MATLAB R2018a.

In this approach, we have chosen not to incorporate tortuous capillaries, but rather to validate the basic network structure before adding any additional complexity. For a fair comparison tortuous lengths were ignored in the anatomical networks and instead straight vessel lengths were computed directly as the distance between each pair of connected vertices.

Similarly, although a Gaussian distribution of capillary diameters has been reported (6.23±1.3μm in humans; Cassot et al., 2006), we have not attempted to assign physiological diameters. To do so would be a complex task due to possible variations along arteriolar-venular flow pathways, local parent–daughter correlations, and imaging uncertainties (e.g., shrinkage of vessels; Tsai et al., 2009; Steinman et al., 2017; Di Giovanna et al., 2018). Instead, uniform diameters of 5μm were imposed in all synthetic, anatomical, and lattice networks.

##### 2.2.1.3. Pruning the network

Details of these steps are given in section S1.2. of the Supplementary Material. Throughout, vertex indices were randomized to avoid any anisotropy arising from deleting vertices or edges in a preferential order. Firstly, by considering each polyhedron of the Voronoi diagram in turn, very small or narrow polyhedral faces were merged with neighboring faces, which greatly reduced the vessel density (Figure 2C). Despite no longer strictly defining a Voronoi tessellation according to the initial distribution of seed points, the distribution of polyhedral volumes remained Gaussian with mean approximately $L_{C}^{3}$ (Figure 2C, inset), analogous to the distribution of polygonal areas in the 2D case.

Next, pairs of closely-located vertices (less than a specified distance apart, see section S1.2. and Figures S1a-c) were identified and merged, thus reducing the vertex density and the number of very short capillaries. Excess edges were deleted, with the criterion that neighboring vertices still had at least three connecting edges. For this reason some vertices with more than three connections may remain because all their neighboring vertices had only three connections. These vertices were finally split into multiple bifurcations (section S1.3 and Figures S1d-g).

A smaller ROI was extracted from a larger network in order to avoid boundary effects (section S1.4). For a fair comparison, synthetic networks were generated with equal dimensions to the relevant anatomical (mouse or human) ROIs. A final check for close-lying vertices was performed, and vertices merged/removed if necessary. At this stage, a small percentage of multiply-connected vertices with >3 connections may arise (quantified in the Results). The final network data was written in the standard Avizo ASCII format, generating 10 networks for each set of parameter values studied.

#### 2.2.2. Simple Grid-Like Lattice Networks

Two types of simple lattice networks were generated following (Peyrounette et al., 2018); their elementary motifs are shown in Figure 1D. Both of these networks are by design periodic, isotropic and homogeneous.

The cubic lattice network (CLN) is a regular 3D cubic grid with side length *L* and 6-connectivity.

The periodic lattice network (PLN) is also composed of a periodically repeating motif but with 3-connectivity, a characteristic topological feature of cerebral capillaries (see section 3.3.3). Thus, it is expected that this PLN will more closely mimic the anatomical and synthetic networks than the CLN. This network was generated by connecting regularly-placed cubes of side length 2*L* with one capillary link of length 0.5*L* on each edge of the cube, inspired by the simple foam model of Gibson and Ashby (1982).

By analogy with the characteristic length *L*_*C*_, defined above as the length of the cells used to constrain the Voronoi diagrams, we use here *L*_*C*_ to refer to the length of the elementary motifs in lattice networks, thus *L*_*C*_ = *L* in the CLN and *L*_*C*_ = 3*L* in the PLN.

### 2.3. Definition of Quantitative Metrics for Characterizing Cerebral Capillary Networks

Next, we define the quantitative metrics used in combination to characterize and compare capillary networks. These metrics can be classified into two types: the architectural metrics asses their space-filling nature (section 2.3.1), morphology (section 2.3.2) and topology (section 2.3.3). The functional metrics asses flow (section 2.3.4), blood/tissue exchange (section 2.3.5) and robustness to capillary occlusions (section 2.3.6). Many of these metrics have been previously used to analyze capillary networks. Others ones are inspired from other fields, e.g., porous media physics (section 2.3.5) or constitute novel additions to the literature (section 2.3.6).

#### 2.3.1. Space-Filling Nature of Capillary Networks

A key feature of cerebral capillary architecture that we wish to replicate in the synthetic networks is that they are homogeneous i.e., space-filling at scales above a cut-off length of 25–75 μm (Lorthois and Cassot, 2010). In contrast, arterioles and venules are quasi-fractal and scale-invariant (Cassot et al., 2009; Lorthois and Cassot, 2010). Following (Lorthois and Cassot, 2010), the non-fractal, space-filling nature of the capillary networks in all ROIs was tested via a multiscale box-counting analysis of the local maxima of extravascular distances (EVDs), see Appendix A.1.

Additional metrics were extracted from the EVDs, starting with the mean EVD and the mean of the local maxima, i.e., the mean of EVD values computed for all local maxima. The EVD is also related to mass transfer properties, which are strongly dependent on the local spatial arrangement of the capillaries, among other factors (see section 2.3.5). Indeed, Baish et al. (2011) showed that both the maximum EVD and the “convexity index” reveal distinct properties for tumor vs. healthy networks. The convexity index was defined as the slope of a linear fit to the log-log scale histogram of EVDs at small scales (Appendix A.1). Baish et al. showed that the maximum EVD was inversely (non-linearly) correlated to the convexity index. Here, both metrics were calculated.

#### 2.3.2. Morphometrical Metrics

The following metrics were computed to quantitatively compare the morphometrical properties of networks:

1. Distribution, mean and SD of vessel lengths,
2. Edge density (number of vessels per volume),
3. Length density (sum of vessel lengths per volume),
4. Interior vertex density (number of non-boundary vertices per volume),
5. Boundary vertex density (number of boundary vertices per surface area of the region of interest).

#### 2.3.3. Topological Metrics

For a simple topological metric, the percentage of interior vertices with more than three connections was calculated.

For a more thorough quantitative assessment, an algorithm to identify the shortest loops in a network was developed.

**Figure 3:**
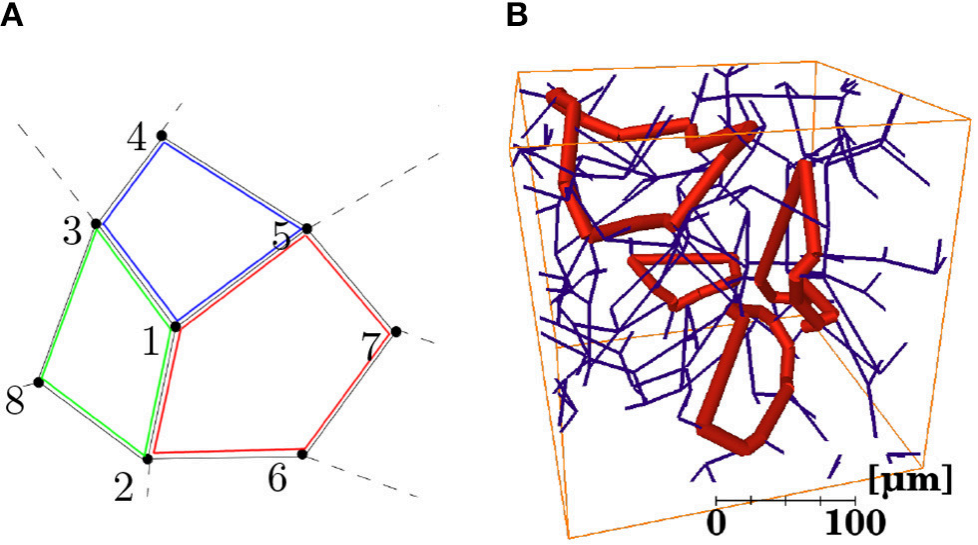
**(A)** Schematic illustration of a section of network containing three capillary loops (identified in red, green, and blue) centered around a “root vertex” labeled *1*. **(B)** Four individual loops (in thick red) identified in a synthetic network (in dark blue).

The shortest loops associated with each vertex *v*_*i*_ were defined as the set of closed loops starting at *v*_*i*_ that also pass through neighboring connected vertices $v_{j}^{neigh}$ and $v_{k}^{neigh}$, for all values of *j* = 1,…,*n* and *k* = 1,…,*n*, *k*≠*j*, where *n* is the number of neighboring vertices. The procedure for identifying capillary loops is illustrated in Figure 3A. Identifying the neighbor vertices 2, 3, and 5 directly connected to the root vertex 1, each of the three possible pairs of these vertices was considered in turn. The shortest path between each pair of vertices without passing through the root vertex was computed using Dijkstra's algorithm. Here, each edge was assumed to have unit weight for simplicity, but in practice edges were weighted by their length. The shortest path between vertices 3 and 5 without passing through vertex 1 is 3-4-5. This path was then added to the edges linking vertices 3 and 5 with the root vertex to obtain the final loop path 1-3-4-5-1. For this “root vertex,” two other loops, 1-5-7-6-2-1 and 1-2-8-3-1, were also found. For each root vertex, there are a maximum of *C*_2_(*n*) loops, where *n* is normally 3. However, each loop was identified multiple times (once for each vertex in the loop) and repetitions were identified and deleted. Selected loops identified in a synthetic network are shown in Figure 3B. The mean number of edges per loop, mean total loop length and mean number of loops per edge were calculated for all networks.

#### 2.3.4. Flow Metrics

As discussed in section 2.2.1, for simplicity, uniform vessel diameters of 5μm were assigned in all ROIs for the purpose of blood flow simulations. Flow solutions were computed using an in-house 1D network flow solver (Peyrounette et al., 2018), which takes a classical network approach i.e., assumes a linear relationship between flow and pressure drop in vessels, and conservation of flow at vertices (Appendix A.2). For brevity, all flow results are presented for a pressure gradient in the *x*-direction only.

The velocity in each capillary was calculated by dividing the flowrate by the vessel cross-section, and the mean and SD of velocities in each ROI was computed.

Next, the permeability was computed. This effective parameter captures the capacity for blood to flow through a representative portion of the network. If divided by the effective viscosity, it is sometimes referred to as the network conductance (Smith et al., 2014; El-Bouri and Payne, 2015). Following (Reichold et al., 2009), the permeability was calculated by applying a pressure gradient across the ROI. By analogy with the theoretical value obtained by applying volume-averaging/homogenization techniques to derive Darcy flow (Smith et al., 2014), the permeability is then given by:

(1) $$\begin{array}{r} K_{x}=\frac{\mu }{\Delta P_{x}/L_{x}}\frac{Q_{x}}{A_{x}}, \end{array}$$

where *K*_*x*_, Δ*P*_*x*_, and *L*_*x*_ are the permeability, pressure drop and length of the domain, respectively, in the *x*-direction. *Q*_*x*_ is the corresponding global flowrate, defined as the sum of the flows entering the domain through the face perpendicular to the *x*-direction, and *A*_*x*_ is the area of this face. Because all diameters are uniform, the effective viscosity μ is simply the viscosity in all vessels. Note that in contrast to the velocity, the permeability is independent of the magnitude of Δ*P*.

#### 2.3.5. Mass Transfer Metrics

Firstly, the transit time (i.e., the time spent by blood traversing each capillary) was calculated as the vessel length divided by the mean vessel velocity, to yield the distribution of transit times, and the median transit time was recorded.

Secondly, in a similar way to the permeability calculation, averaging techniques were employed to derive a macro-scale effective parameter *h*, known as the mass exchange coefficient (Whitaker, 1999). For details of this method see Appendix A.3. This coefficient captures the network-specific capability for mass transfer between the capillaries and the surrounding tissue. The value of *h* characterizes the network architecture and the diffusion properties of both blood and tissue. Here, we consider the diffusion of a non-reactive, non-metabolic tracer, which is highly diffusible through the blood brain barrier. Under these assumptions, and for space-filling networks, *h* is correlated with the surface area available for mass exchange and hence also with the vessel length density, given the uniform distribution of diameters assigned here. The mass exchange coefficient *h* is reported for a ratio of tissue to vessel diffusion coefficients of 0.25 (Appendix A.3).

#### 2.3.6. Robustness to Occlusions

The robustness of the capillary networks to occlusions was quantified by applying a single occlusion in turn to each edge upstream of a three-connected vertex. Numerically, occlusions were imposed via a diameter reduction factor of 100 in the occluded edge (Cruz Hernández et al., 2019). Because of their different behavior, converging (two inflows, one outflow) and diverging (one inflow, two outflows) vertices were considered separately as in Nishimura et al. (2006). The ratio of post- to pre-occlusion flowrates in the outflow edge(s) was computed, with the criterion that baseline i.e., pre-occlusion absolute flowrates in all inflow and outflow edges were greater than a specified tolerance (*q*_tol_ = 0.001% of the total inflow), otherwise the edge was ignored. The final metric reported was the mean of these flow ratios for each case, averaged over all ROIs.

## 3. Results

In this section, we first assess the architecture of mice and humans capillary networks using the simplest morphometrical and topological metrics. As wee shall see in section 3.1, the results suggest that rescaling is needed to accurately compare capillary networks between species. This implies that the characteristic length *L*_*C*_ of the synthetic networks developed here needs to be independently chosen for both species. To guide this choice, we study their scaling properties, as well as those of the simpler grid-like networks, as a function of domain size and *L*_*C*_ in section 3.2. Finally, the structure and function of these networks with *L*_*C*_ = 75μm and *L*_*C*_ = 95μm is compared to those of the mouse and human data in sections 3.3 and 3.4, respectively.

### 3.1. A Simple Re-scaling Accounts for Inter-species Differences in Anatomical Networks

**Figure 4:**
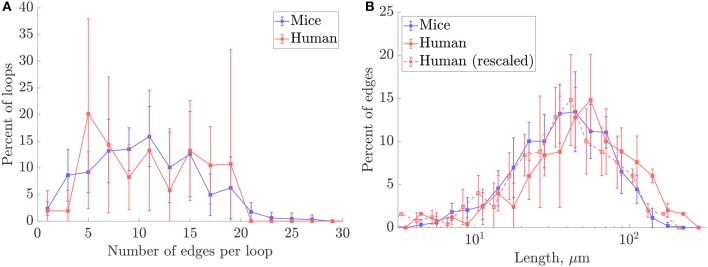
Histograms of **(A)** number of edges per loop, and **(B)** capillary lengths on a log-scale, in mouse and human ROIs. The human length distribution rescaled to match the mean length for mice is superimposed in dashed lines. For all plots, frequencies were collected over all ROIs for each species. Error bars show the SD between ROIs.

A preliminary comparison between the mouse and human anatomical networks was conducted using the simplest morphometric metrics (Table 1, Table S1). These showed that capillaries in the human ROIs were longer (mean capillary length 34.4% higher) and spaced further apart (mean EVD 15.8% higher) than in mice. Nonetheless, loop metrics were very similar, with the mean number of edges per loop almost identical between species. The histograms of this metric were also similar, although with more variance for humans (Figure 4A) suggesting that this distribution was not statistically converged with *N* = 4 samples (compared to *N* = 7 for mice). Thus, the underlying topology of the networks is comparable but the scaling of the human network is increased relative to the mouse.

**Table 1:** The geometrical, topological and functional metrics calculated here, for mice, synthetic with *L*_*C*_ = 75μm (“S75”), and lattice ROIs.

**Metric**	**Mice**	**S75**	**Periodic Lattice**	**Cubic Lattice**
***N***	**7**	**10**	**1**	**1**
Mean EVD (μm)	18.4 ± 0.9	20.2 ± 0.6	18.9	19.3
Mean local max EVD (μm)	29.4 ± 1.5	34.5 ± 1.4	36.0	47.4
Max EVD (μm)	50.1 ± 3.7	53.4 ± 2.6	57.3	47.4
Convexity index	0.9 ± 0.1	0.9 ± 0.0	0.8	0.8
Mean length (μm)	44.8 ± 2.4	36.0 ± 1.5	41.0	67.0
SD length (μm)	28.1 ± 2.3	18.5 ± 1.5	0.0	0.0
Edge density (10^3^ mm^−3^)	17.0 ± 1.4	21.3 ± 0.8	17.7	12.5
Length density (mm^−2^)	673 ± 58	674 ± 20	661	668
Vertex density (10^3^ mm^−3^)	8.2 ± 0.6	11.4 ± 0.4	10.7	3.3
Boundary vertex density (mm^−2^)	351 ± 46	317 ± 23	132	223
% multiply-connected vertices	7.2 ± 0.9	2.2 ± 1.0	0.0	100.0
Mean no. edge/loop	11.2 ± 1.2	10.3 ± 0.6	9.0	5.1
Mean loop length (μm)	486 ± 60	368 ± 35	369	345
Mean no. loop/edge	5.1 ± 0.3	4.9 ± 0.4	4.0	9.0
Mean velocity (μm/s)	197 ± 43	204 ± 29	286	268
SD velocity (μm/s)	258 ± 31	233 ± 18	273	380
Permeability (10^−3^μm^2^)	1.57 ± 0.38	1.38 ± 0.26	2.03	3.42
Median transit time (s)	0.14 ± 0.04	0.13 ± 0.02	0.10	0.08
Exchange coefficient *h*	24.9 ± 3.31	21.3 ± 0.83	31.5	33.1
Post-occlusion flow ratio (converging)	0.77 ± 0.01	0.76 ± 0.01	0.69	-
Post-occlusion flow ratio (diverging; branch A)	0.26 ± 0.03	0.29 ± 0.02	0.07	-

*Results are presented as mean ± S.D. over the N ROIs studied for each network type (i.e., for the metric “Mean length,” the mean length was calculated for each ROI, and the mean and S.D. of these mean lengths over all ROIs are presented in the table). Colors indicate values that are within 10% (green), more than 10% lower (blue) or more than 10% higher (red) than the corresponding values for the mice ROIs. Permeabilities, velocities and transit times were calculated with uniform diameters of 5μm. Some key metrics are represented (as percentage errors relative to values for the mice data) in Figure 10*.

This hypothesis was supported by down-scaling the human capillary lengths by the cross-species difference in mean lengths. The rescaled length histograms for humans (red dashed lines in Figure 4B), coincided closely with the histogram for mice. Thus, we hypothesize that the synthetic networks developed here can be generated to model either mouse or human cerebral capillary networks by an appropriate choice of characteristic length *L*_*C*_ for each species.

### 3.2. Scaling and Convergence of Metrics in Synthetic Networks

Metrics characterizing the architectural, flow and transport properties of porous or heterogeneous media usually vary with the size of the domain under study until a characteristic size is reached, known as a Representative Elementary Volume (REV) (Bear, 1988). Above this REV size, the medium can be considered homogeneous and finite-size effects become negligible. Here, convergence of properties of the synthetic networks with domain size is first studied to determine their REV. This enables overcoming the difficulty associated to anatomical datasets, where both arterioles/venules and capillaries are intermingled, which makes it only possible to extract capillary regions of limited size, may be smaller than the REV. The scaling properties of the synthetic networks with *L*_*C*_ are then investigated. For that purpose, some metrics were normalized by an appropriate power of *L*_*C*_, guided by the derivation of analytical expressions for these metrics in the lattice networks, which was possible thanks to their simple architecture. As detailed in section S2.1 of the Supplementary Material, the mean loop length, length density, and permeability scaled with *L*_*C*_, $1/L_{C}^{2}$, and $\frac{d^{4}}{L_{C}^{2}}$, respectively, where *d* is the vessel diameter.

#### 3.2.1. Convergence of Metrics With Domain Size

**Figure 5:**
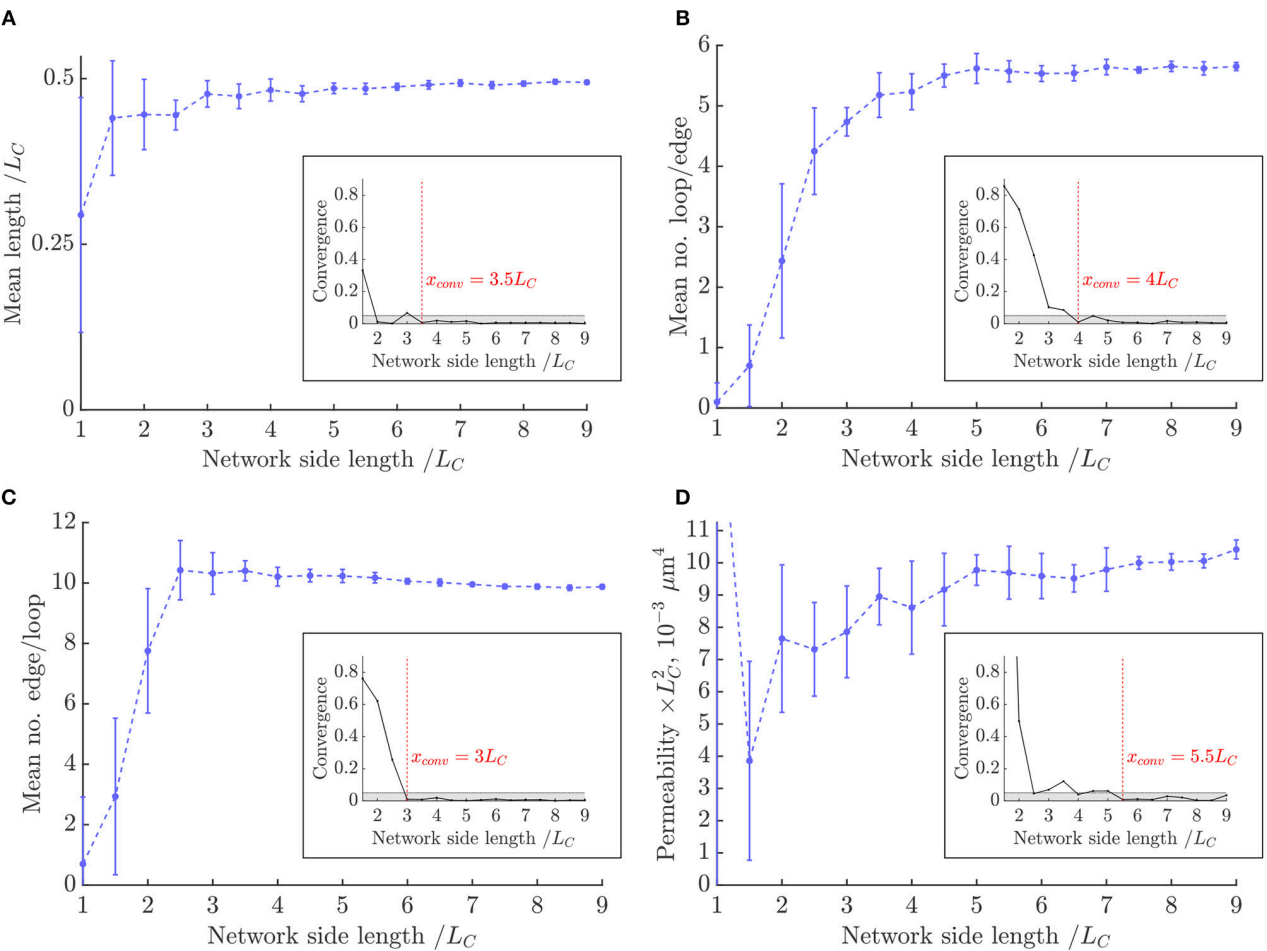
Convergence of metrics with domain size in the synthetic networks: **(A)** mean length, **(B)** mean number of loops per edge, **(C)** mean number of edges per loop, **(D)** mean permeability. Metrics were normalized by the appropriate power of *L*_*C*_. Insets: the convergence of each metric as defined in Equation (2). The converged size *x*_*conv*_ is the size from which the convergence was < 0.05.

The convergence of metrics in the synthetic networks was studied for domain sizes from $L_{C}^{3}$ to ${\left(9L_{C}\right)}^{3}$, with metrics normalized by the appropriate power of *L*_*C*_ (Figure 5).

The convergence of metrics was defined as:

(2) $$\begin{array}{r} \frac{M^{k}-M^{k-1}}{M^{k}}, \end{array}$$

where *M*^*k*^ is the value of the metric in question at size *k*. Each metric was considered converged once this value was < 0.05. The convergence plots of loop metric with domain size are shown in the insets of Figure 5 and Figure S4. Loop metrics in particular were highly sensitive to finite-size effects, as expected from the analytical results obtained in the lattice networks (section S2.1). For example, the number of loops per edge was higher in vessels nearer the center of the domain than near the boundary (Figure 9A in Results), explaining the dependence of this metric on domain size.

The mean length, mean number of edges per loop and mean number of loops per edge all converged for domain sizes between ${\left(3L_{C}\right)}^{3}$ and ${\left(4L_{C}\right)}^{3}$. This is much faster than in the lattice networks (Figure S2), suggesting that the introduction of randomness to network structures reduces the sensitivity of loop metrics to finite-size effects. By contrast, the permeability converged slower, by sizes of ${\left(5.5L_{C}\right)}^{3}$.

This is slower than the results recently presented by our group (Peyrounette et al., 2018), where a range of network sizes were obtained by extracting sub-regions from the largest network studied; in contrast, here networks were stochastically re-generated independently for each size, leading to more variance. Interestingly, the permeability converged immediately in the lattice networks (section S2.1), showing that simple lattice networks cannot be used as an analogy to define appropriate REV sizes for more disordered Voronoi-like networks.

In these networks, for all the considered metrics to converge to within 5%, the domain size should be at least ${\left(5.5L_{C}\right)}^{3}$, which defines the size of the REV.

Above, e.g., with a domain size of ${\left(9L_{C}\right)}^{3}$, the mean vessel length converged to 0.49*L*_*C*_, while the mean number of edges per loop and loops per edge converged to 9.9 and 5.7, respectively. The mean permeability converged to $10.4/L_{C}^{2}\mu$m^4^ with vessels of diameter 5μm, or $0.017d^{4}/L_{C}^{2}$.

#### 3.2.2. Scaling With Characteristic Length *L*_*C*_

**Figure 6:**
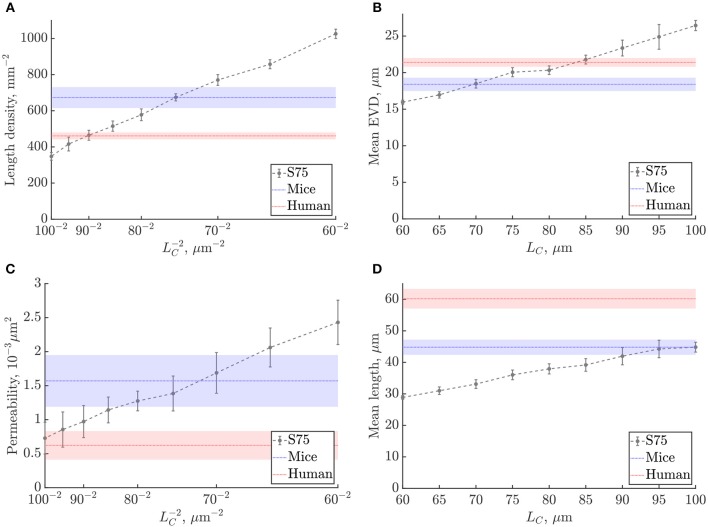
Scaling of metrics with the characteristic length *L*_*C*_ in the synthetic networks: **(A)** length density, **(B)** mean EVD, **(C)** permeability, **(D)** mean length. Errorbars show mean ± S.D. for the synthetic networks. Shaded bands in blue and red show mean ± S.D. of mouse and human values, respectively.

The scaling of metrics was studied for *L*_*C*_ between 60μm and 100μm, with fixed domain size (240μm)^3^ corresponding to the size of the mouse ROIs (Figure 6, Figure S5). As expected from the lattice networks (section S2.1), mean capillary length, mean EVD and mean loop length were linearly proportional to *L*_*C*_, while length density and permeability both scaled with $1/L_{C}^{2}$. For reference, linear fits to these graphs are given in Table S2. The mean number of edges per loop did not change with *L*_*C*_ for the range of values considered (Figure S5c), which is not surprising since this is a purely topological metric.

We chose to derive appropriate values of *L*_*C*_ by matching the length densities in the synthetic networks and the anatomical data. Since uniform diameters were imposed in all networks, the length density is linearly proportional to both the porosity i.e., volume fraction of the domain occupied by vessels, which is important for the flow properties of the network, and also to the vessel surface area per volume, which is a key determinant of mass transfer properties. To best match the mean length density in the mouse ROIs, we chose *L*_*C*_ = 75μm, while for humans we set *L*_*C*_ = 90μm (Figure 6A). By matching length density, we obtain a compromise between matching mean length and mean loop length, which were too low, and the mean EVD and edge density which were too high. With these choices of *L*_*C*_, the mean permeability was lower than mice and higher than humans, but nonetheless fell within or just outside the error bands for both species. The SD was particularly high for the permeability and of the same order for synthetic and anatomical networks (Figures 6C).

Since this study was conducted with variable *L*_*C*_ at a fixed domain size, the number of unit cells decreases with increasing *L*_*C*_, possibly introducing finite-size effects. In the range considered, the number of cells varied from 4^3^ with *L*_*C*_ = 60μm to 2.4^3^ with *L*_*C*_ = 100μm. The decrease in the mean number of loops per edge as a function of *L*_*C*_ (Figure S5d) demonstrates this effect: as shown in the previous section, this metric converges from 4^3^ unit cells, and does not depend on *L*_*C*_ for larger domain sizes. Nonetheless, the length density converged very quickly with domain size, for 2^3^ unit cells or more (Figure S4b), thus the choice of *L*_*C*_ via the length density was unaffected by finite-size effects. Finite-size effects also had a small influence on the linear fits shown in Table S2; if keeping the number of cells fixed to e.g., 3^3^, a maximum difference of approximately 14% was found in the predicted slope.

Final synthetic networks were thus generated in the same domain sizes as the corresponding anatomical ROIs. Synthetic networks matched to the mouse data had domain size (240μm)^3^; with *L*_*C*_ = 75μm, this size is equivalent to ${\left(3.2L_{C}\right)}^{3}$, or (0.58)^3^× the REV size. The error in the calculated metrics due to the finite domain size was estimated using the previous convergence study. For example, the number of edges per loop converged quickly with increasing domain size, and, in the ROI sizes studied, was predicted to deviate only 4% from the converged value. However, the predicted permeability with ROIs of (240μm)^3^ was expected to be approximately 25% lower than its converged value. REV sizes and corresponding convergence trends could not be determined for the anatomical datasets, due to the limited size of capillary ROIs. However if we assume that metrics converge in a similar way, similar finite-size related errors can be expected.

Similar to the synthetic networks, the lattice networks were scaled to match the mean length density in the anatomical networks, to minimize any differences due to scaling. However, as lattice networks did not have equivalent properties either to mice or humans, results for the lattice networks scaled to match the mouse data only are presented in section S2 of the Supplementary Material.

### 3.3. Synthetic Networks With *L*_*C*_ = 75μm Effectively Replicate Mouse Capillary Networks

Metrics computed for synthetic networks with *L*_*C*_ = 75μm and domain size (240μm)^3^ were compared to their values in the mouse networks. The mean and SD across all ROIs of all metrics are listed in Table 1.

#### 3.3.1. Space-Filling Metrics: Synthetic Networks Have Equivalent Space-Filling Properties as Mice ROIs

**Figure 7:**
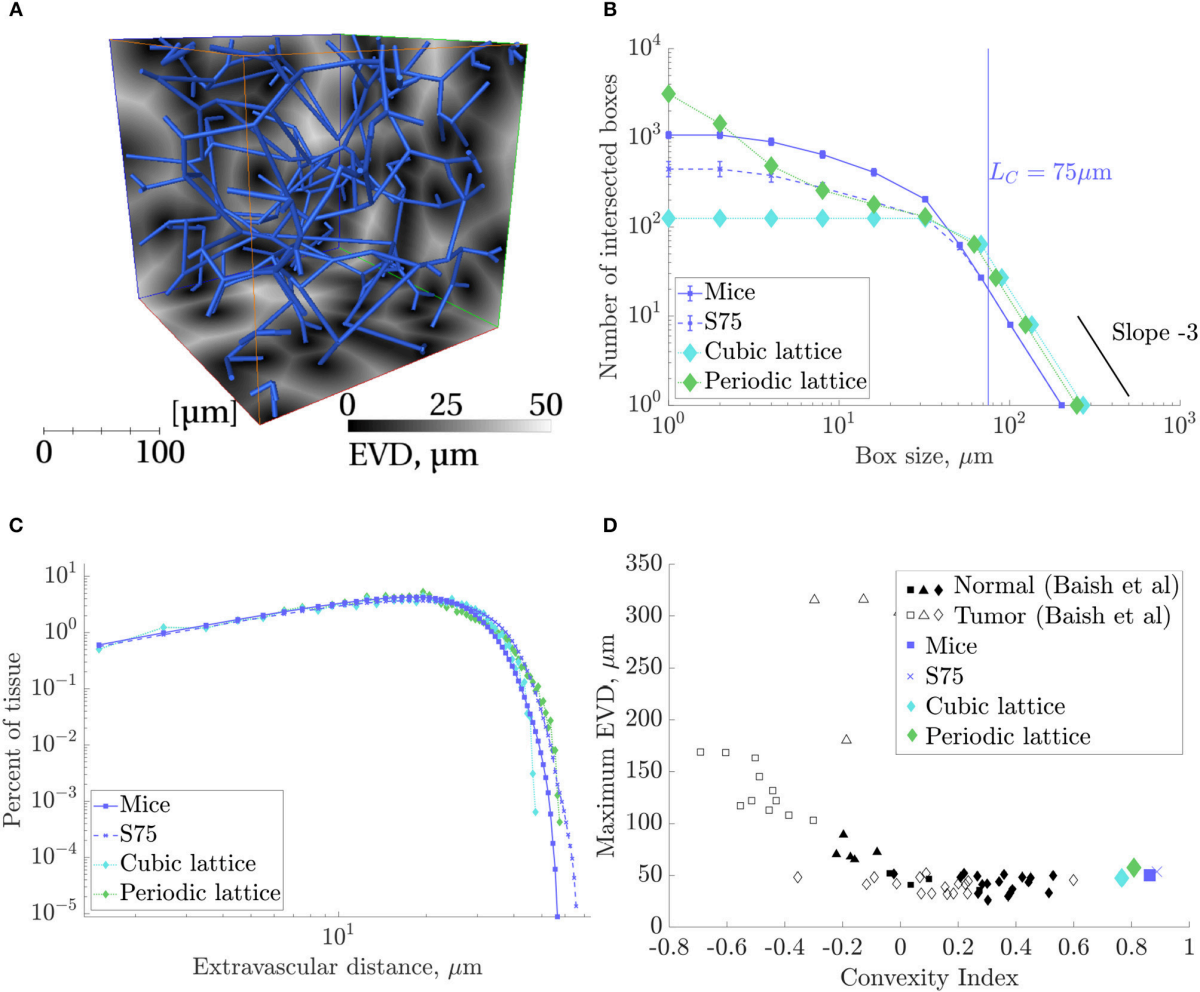
Results of space-filling metrics for the mouse ROIs, synthetic networks with *L*_*C*_ = 75 and domain size (240μm)^3^ (“S75”), and lattice networks, collected over all ROIs. **(A)** A S75 synthetic network in blue, superimposed on three cross-sections of the corresponding EVD field in grayscale, computed for voxels of size 1μm^3^. **(B)** Results of a box counting analysis of the local maxima of EVDs: the number of boxes containing at least one local maxima, *N*(*r*), against box size, *r*. For boxes approximately equal to *L*_*C*_ = 75μm and above, the slope converged to −3. **(C)** Histogram of EVDs on a log-log scale. **(D)** Maximum EVD against the convexity index defined by Baish et al. (2011). Normal and tumor network data points taken from Baish et al. (2011).

Slices of the EVD map with the corresponding synthetic network superimposed are shown in Figure 7A. Applying box-counting methods to the local maxima of EVDs confirmed the homogeneous i.e., space-filling nature of the synthetic networks as well as that of the mouse networks studied (Figure 7B). Lattice networks are also shown for reference. There was no linear domain but rather a continuous variation in slope, characteristic of 3D space-filling structures, until reaching a slope of −3 for scales on the order of *L*_*C*_ or larger.

The mean EVD in the synthetic ROIs was slightly (<10%) higher than in the mouse ROIs, while the mean of the local maxima of EVDs was 17% higher. The histograms of EVD on a log-log scale (see Figure 7C) also showed a similar distribution between all networks, including the lattice networks.

Convexity indices were very close, and the maximum EVD was between 47 and 58μm for all networks (Figure 7D). Both metrics were well within the range of what was classified as “normal” rather than “tumorous” by Baish et al. (2011).

#### 3.3.2. Morphometrical Metrics: Length Densities Were Well-Matched but Mean Lengths Were Lower in Synthetic Networks

**Figure 8:**
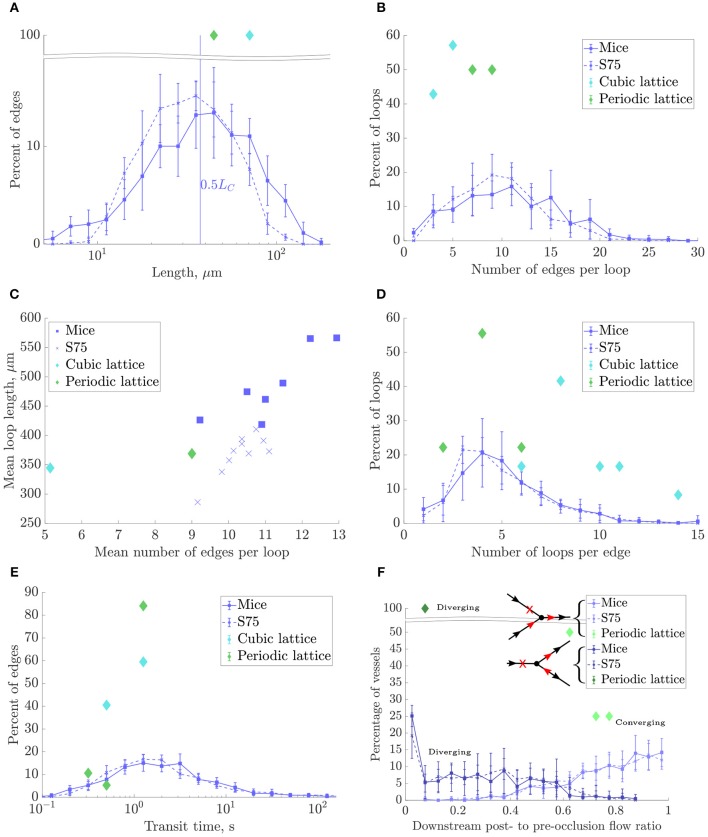
Morphometrical, topological and functional results for the mouse ROIs, synthetic networks with *L*_*C*_ = 75 and domain size (240μm)^3^ (“S75”), and lattice networks. In all plots except **(C)**, data points represent the mean over all ROIs for each network type, and errorbars indicate the SD between ROIs. **(A)** Histogram of lengths on a log-scale. **(B)** Histogram of number of edges per loop. **(C)** Mean loop lenght, μm, vs. mean number of edges per loop for each ROI. **(D)** Histogram of number of loops per edge. **(E)** Histogram of capillary transit times, on a log-scale. **(F)** Histograms of post- to pre-occlusion absolute flow ratios in vessels one branch downstream from the occlusion, where the vertex downstream of the occlusion has 3-connectivity, and divided into converging and diverging bifurcations as illustrated in the schematics. In the diverging case, flow ratios are plotted for the outflow branch without change in flow direction post-occlusion (branch A). The CLN does not appear because all its vertices had connectivity greater than three.

The log scale distribution of straight vessel lengths collected over all ROIs was qualitatively similar in the synthetic networks to that of mice (Figure 8A). However, mean vessel lengths in the synthetic networks were overall 19.4% lower while the SD was 34% lower (Table 1).

As discussed above, *L*_*C*_ was chosen to match length densities between synthetic and mouse ROIs. Due to the shorter mean capillary length, this resulted in a 25% higher edge density in the synthetic networks than the mice. Similarly, the vertex density was higher (≈39%) in the synthetic networks, while the boundary vertex density was similar (<10% fewer in the synthetic ROIs).

#### 3.3.3. Topological Metrics: Synthetic Networks Had Very Close Loop Topology and Distribution

There were fewer multiply-connected interior vertices in the synthetic networks compared to the mice networks. The mean number of edges per loop compared well, and the distributions were very similar (Figure 8B). An early topological study in the rat cerebral cortex found lower values for this metric (between 4 and 7 capillaries per loop), perhaps due to the difficulty of manually tracing long loops, or species differences (Hudetz et al., 1988). Consistent with the relatively small heterogeneity of vessel length, loop lengths were correlated with the number of edges per loop (Figure 8C) but were on average 24% lower in the synthetic networks, consistent with the lower mean vessel length. The mean number of loops per edge also compared well with mice (within 3%) and the distributions matched very closely (Figure 8D). Both the mean number of edges per loop and loops per edge were independent of *L*_*C*_, and show that the underlying network topology was very well matched between synthetic and mouse networks.

#### 3.3.4. Flow Metrics: Synthetic Networks Had Slightly Higher Permeability

**Figure 9:**
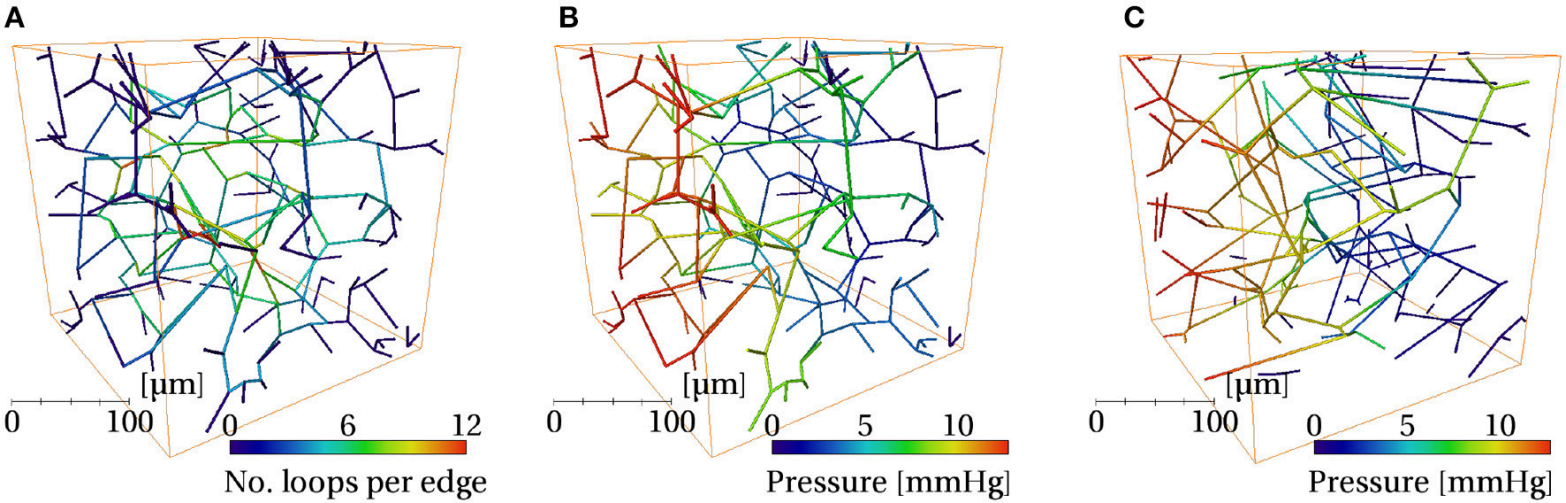
Visualizations of one synthetic ROI with *L*_*C*_ = 75μm **(A,B)** and one mouse ROI **(C)**, both of size (240μm)^3^, color coded by the following quantities: **(A)** number of loops per edge, **(B,C)** pressure.

The simulated pressure distributions are visualized in synthetic and mouse networks in Figures 9B,C, and showed a qualitatively similar distribution. With a pressure gradient in the *x*-direction, the mean blood velocity in the synthetic networks was very close to that in mice. The mean permeability in the synthetic networks was 12% lower. It was verified that for a large number of samples (e.g., *N* = 500), the distribution of permeability values was Gaussian.

#### 3.3.5. Mass Transfer Metrics: Synthetic Networks Had Slightly Lower Exchange Coefficient

The distribution of capillary transit times were very similar between synthetic and mouse ROIs (Figure 8E), as were the median transit times. For all networks, the exchange coefficient *h* followed a linear relationship with *D*_ratio_, the ratio between tissue and vessel diffusion coefficients (Appendix A.3). With *D*_ratio_ = 0.25, *h* was 14% lower in the synthetic vs. mouse networks.

#### 3.3.6. Robustness Metrics: Synthetic and Mouse Networks Were Similarly Robust to Occlusions

Three-connected vertices were split approximately evenly into two cases: converging (2 inflows, 1 outflow) and diverging (1 inflow, 2 outflows), each with distinct behavior due to their specific configurations.

In the converging case, the flow in the outflow branch necessarily decreased post-occlusion and did not change direction, leading to post- to pre-occlusion flow ratios between 0 and 1. Since in this case only one of two inflows was cut, the mean flow reduction was moderate (approximately 25%).

In the diverging case, the post-occlusion flowrate was of equal magnitude in both outflow branches due to mass conservation, and reversed in one branch (branch “B”). This yielded a flow ratio between 0 and 1 in branch “A” and a negative (or zero) flow ratio in branch “B.” In 70–75% of cases for the mice and synthetic ROIs, branch A had the higher pre-occlusion flow, while in 13–18% of cases the post-occlusion flow in both branches was zero. Since in this case the only inflow was blocked, the flow reduction in branch A was much more significant on average (70–75%) than for the converging case.

The distributions of flow ratios for both converging and diverging (branch A only) cases were almost superimposed for synthetic and mice networks (Figure 8F), and the mean flow ratios were also very close (Table 1).

### 3.4. Synthetic Networks With *L*_*C*_ = 90μm Compared to Human ROIs

Results for the synthetic networks with *L*_*C*_ = 90μm and size 264 × 264 × 207μm^3^ compared to humans were very similar, although the agreement was not as good (Figures S6 and S7). This may be partly because fewer human ROIs were extracted (4, rather than 7 for the mouse), thus metrics were less statistically converged in terms of the number of samples. The regions were also smaller in the *z*-direction, although larger in the other two directions. With the larger *L*_*C*_, the ROI size was equivalent to 2.93 × 2.93 × 2.3*L*_*C*_, further from the REV size than the synthetic networks matched to the mouse ROIs, and thus more susceptible to finite-size effects. Key results are discussed next, while complete results of the mean and SD across all ROIs are found in Table S1.

The mean and maximum EVD were 13 and 19% longer, respectively, in the synthetic ROIs than the humans. The mean length was 30% lower, while the edge density was 42% higher. The density of boundary vertices was close, and there was a similar percentage of multiply-connected vertices. There were slightly fewer edges per loop in the synthetic vs. humans, although loop results in the human ROIs were noisy (Figure S7b). Similar to the mean length, the mean loop length was 32% lower in the synthetic ROIs. There were 42% more loops per edge in the synthetic networks, although again the frequency distribution for humans in Figure S7d was not statistically converged. These metrics indicate that the synthetic networks were more closely inter-connected than the human ROIs. This was confirmed by the flow metrics: the mean velocity and permeability were 36 and 39% higher, respectively. In terms of mass transfer, the median transit time was 26% lower, while the mass exchange coefficient *h* was 43% lower than in humans. Finally, when subject to occlusions, the mean post- to pre-occlusion flow ratio was very close between the synthetic and human ROIs, and the distributions of flow ratios in converging and diverging bifurcations were also similar (Figure S7f).

## 4. Discussion

Although the capillaries are the smallest vessels in the brain, their extremely large surface area allows them to fulfill their key function of supply of oxygen and other nutrients and removal of toxic metabolic waste to/from the tissue. Their crucial role in healthy neurovascular function and robustness to vascular damage in disease is becoming increasingly recognized (Farkas and Luiten, 2001; Shih et al., 2015; Østergaard et al., 2016; Cruz Hernández et al., 2019). However, quantitative anatomical data specifically focused on the spatial organization of cerebral capillary networks are extremely scarce, which has made difficult to identify the minimal organizational principles that underly their structure and function. This has, until now, limited the development of synthetic network models built on such principles and prevented their thorough, quantitative validation.

### 4.1. Summary of Key Results

In this context, the key contributions of this paper were:

- To define a complete range of metrics that can be used in combination for thorough characterization of the structure and function cerebral capillary networks;
- To provide a database of these metrics for healthy mouse and human capillary networks, thereby identifying the similarities and differences in scaling;
- To present a novel method for generating 3D synthetic capillary networks with equivalent properties, based on a few simple organizational principles, which can be scaled depending on the species under study.

Relevant quantitative metrics capturing together the key information for characterizing cerebral capillary networks were identified. Many of these metrics had been previously used to analyze the morphology and flow properties of cerebral capillary networks. To the best of our knowledge, however, the topology of their looping, interconnected structure had not been described in detail, nor their mass transfer properties or robustness to occlusions. In particular, we showed for the first time that differences in scaling play a key role in the comparison of anatomical capillary networks, and that this can be evidenced via scale-independent loop metrics that evaluate topological equivalence. This will be useful in future studies to distinguish between structural differences due to scaling, and those due to more fundamental discrepancies such as vascular rarefaction in pathological scenarios such as stroke, dementia, and Alzheimer's Disease (Cruz Hernández et al., 2019). These metrics will thus facilitate comparison between anatomical data extracted from different samples, cortical depths, brain regions, ages, or species (Farkas and Luiten, 2001).

Moreover, 3D synthetic networks were stochastically generated by exploiting fundamental physiological concepts of the spatial organization of cerebral capillary networks i.e., that the intrinsic spacing of the cerebral capillaries is controlled by the limited diffusion distance of oxygen. Spatially-constrained Voronoi diagrams yielded tessellations that were locally randomized, but with homogeneous properties at the network scale. This approach produced networks that complied with the desired global features i.e., they were three-connected, isotropic, space-filling, and with convex extravascular domains of a characteristic size. Importantly, this simple algorithm was not tuned to match specific anatomical statistics such as length distributions, in contrast to others (e.g., Su et al., 2012). Rather, our model relied on one single important parameter with physiological significance, *L*_*C*_, which controls the size of extravascular domains associated with each Voronoi polygon.

**Figure 10:**
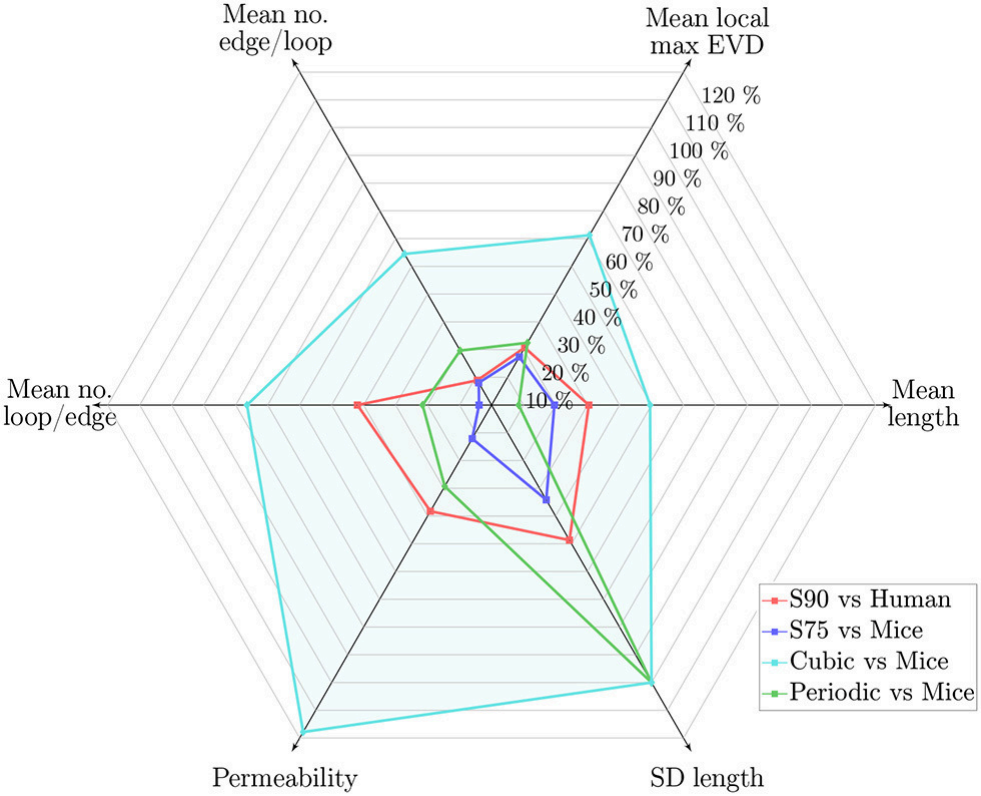
Web chart showing the percentage error for synthetic networks with *L*_*C*_ = 90μm relative to the human ROIs (S90, in red), and *L*_*C*_ = 75μm relative to the mouse ROIs (S75, in blue), and for the PLNs and CLNs vs. the mouse ROIs (in green and turquoise, respectively), for 6 key metrics. Percentage error calculated in terms of the mean of values across all ROIs. Length density error was <2% for all cases.

The characteristic length *L*_*C*_ was chosen by matching the length density in the anatomical ROIs. The resulting difference in key metrics is summarized in Figure 10 for synthetic networks with *L*_*C*_ = 75μm relative to mouse ROIs, and with *L*_*C*_ = 90μm relative to humans. It is clear that the synthetic networks performed better in comparison to the mouse networks than the human, which may be at least in part due to issues with the human dataset (residual imaging artifacts, fewer ROIs, network anisotropy), see section 4.2.

Scaled to the mouse data, the mean vessel length was lower in the synthetic networks, but the mean EVD was slightly higher. Two topological metrics (the mean number of edges per loop and the number of loops per edge) were very close to mice. In terms of functional metrics, the mean permeability was slightly lower, while the mean velocity and median transit times were close. In terms of mass transport and robustness, the mass exchange coefficient was slightly lower in the synthetic networks, while the post-occlusion downstream flow ratios in converging and diverging bifurcations were very close.

In contrast to the Voronoi-like synthetic networks, the lattice networks did not replicate the anatomical networks so well (Figure 10). The CLN performed worst, notably with very high mean length, number of loops per edge and permeability, very few edges per loop, and zero SD of lengths. Errors for the PLN were of a similar order of magnitude to those for the synthetic networks scaled for the human data, except notably the SD of vessel lengths was zero due to its highly ordered construction, leading to a large error relative to the mouse data. This demonstrates that the naive approach of constructing simple grid-like networks was not sufficient to replicate the geometrical or functional properties of cerebral capillary networks, and highlights the need for introducing a sufficient level of randomness in the generation scheme. Nonetheless, the PNL performed surprisingly well, perhaps due to having a similar connectivity to the anatomical networks.

The excellent results in the Voronoi-like synthetic networks show that we have identified the minimal organizational requirements of the cerebral capillary networks which are key to replicating their architectural and functional properties, including flow, transport and robustness to occlusions.

### 4.2. Limitations and Perspectives

The first limitation comes from the limited number of ROIs extracted from both the human and mouse data. All mouse ROIs were at a cortical depth of 650μm or more, to maximize ROI size while avoiding vessels of diameter >10μm (assumed to be the maximum capillary diameter). In this zone, corresponding roughly to layer IV, the capillary network is approximately isotropic; in contrast, we observed more anisotropy near the cortical surface, consistent with previous observations (Duvernoy et al., 1981; Farkas and Luiten, 2001; Cassot et al., 2006). In humans, however, whatever the depth of the ROI (3 out of 4 were at depths >1 mm), the permeability was highly anisotropic: only the permeability in the *x*-direction, *K*_*x*_, was presented in the Results, but preferential alignment of capillaries perpendicular to the cortical surface led to a ≈260% higher *K*_*y*_. In contrast, in the confocal imaging direction, *K*_*z*_ was roughly 80% lower than *K*_*x*_ probably due to signal reduction in the deepest images.

Manual correction of the automatic segmentation of the human data was necessary to remove various artifacts (small capillary loops, broken capillaries indicating loss of network connectivity) present in the original segmentation (Cassot et al., 2006). For the same zones, the newly-segmented networks had 50%, 187% and 76% higher mean vessel length, loop length and permeability *K*_*x*_, respectively, compared to the original segmentation, with a 17% lower edge density (mainly due to the removal of short artifactual edges).

Even with manual correction, there are inevitably errors and artifacts introduced during any image acquisition and processing protocol (e.g., unfilled vessels, sample shrinkage or distortion, low signal-to-noise ratio, artifactual removal or addition of short or small diameter vessels). This means that anatomical data may not be an exact representation of the *in vivo* microvasculature. Promising methods to quantitatively evaluate different segmentations (Mayerich et al., 2012) are nonetheless hindered by the lack of a ground truth. Physiologically-based synthetically-generated networks, combined with models of the artifacts engendered by specific imaging processes, may help quantify the imaging-associated uncertainty inherent in anatomical datasets.

Another limitation comes from the simplified approach taken for generating the Voronoi-like synthetic networks. Previously, even simpler models have been introduced to mimic the capillary bed. For example, infinite single, parallel or randomly-oriented cylinders, have often been used (Baish et al., 2011; Pflugfelder et al., 2011; Jespersen and Stergaard, 2012; Lorthois et al., 2014a), which might lead to flawed estimations of functional properties at the scale of the capillary network. Baish et al. (2011) constructed a range of artificial networks e.g., cylindrical arrays, spherical holes, quasi-fractal structures and randomized networks at the percolation limit, to derive metrics (i.e., the maximum EVD and convexity index calculated here) which differentiate tumor-like from healthy structures, and hence deduce scaling laws for drug delivery times. Here, the convexity metric confirmed that our synthetic networks were representative of healthy as opposed to tumorous tissue. However, since results were very close for all ROIs, including lattice networks, this metric alone could not reliably evaluate the similarity of model networks to anatomical data. Another model (Reichold et al., 2009) employed a regular capillary grid connected to fractal trees to study the effect of capillary dilation on flow and transport.

**Figure 11:**
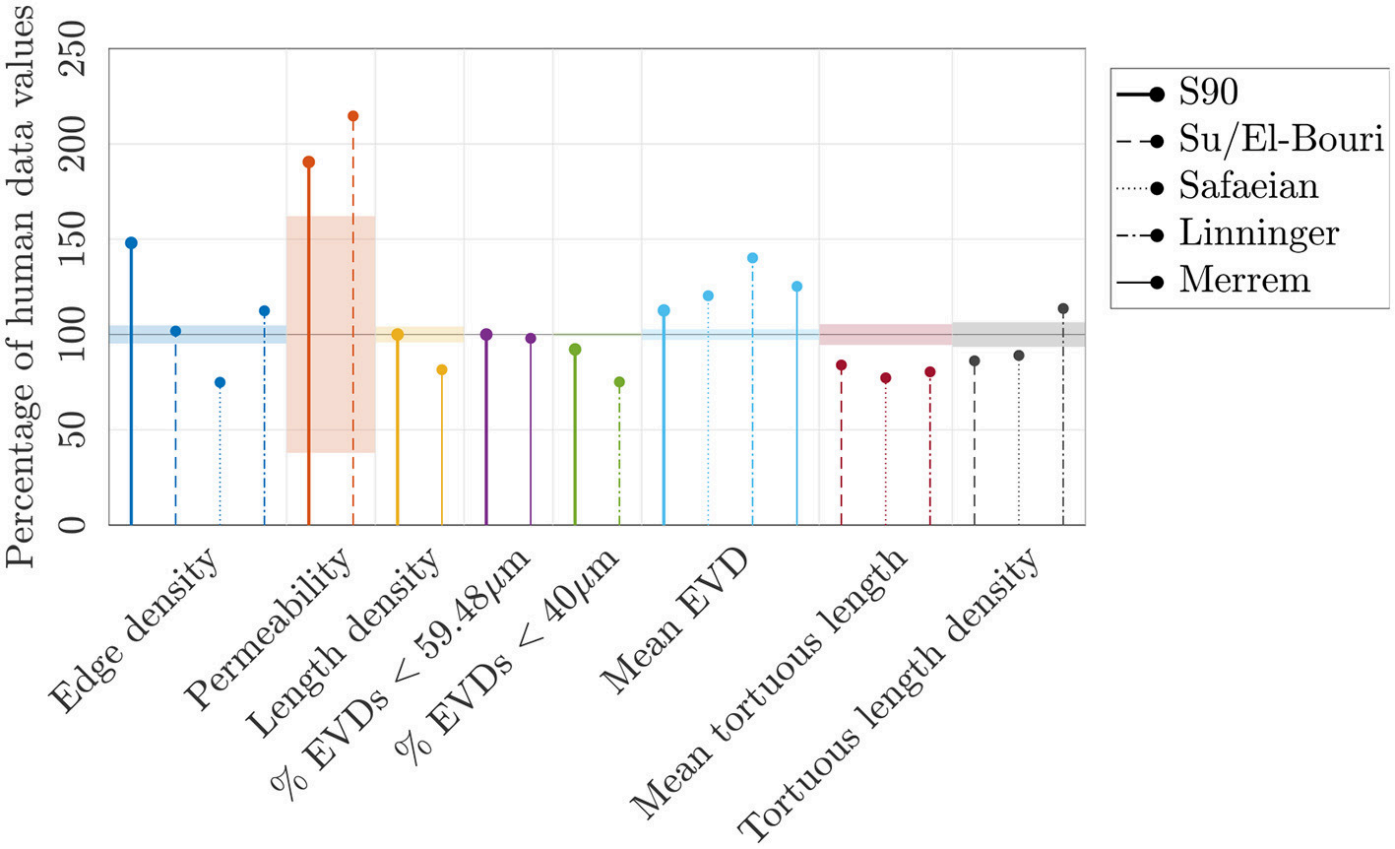
A range of metrics expressed as a percentage of values in the human ROIs, for the synthetic networks with *L*_*C*_ = 90μm (“S90”), and reported by Su et al. (2012), El-Bouri and Payne (2015), Safaeian et al. (2011), Linninger et al. (2013), and Merrem et al. (2017). The permeability in the S90 networks and reported by El-Bouri and Payne (2015) were calculated for *N* = 500 networks in a domain size of (375μm)^3^, with a Gaussian distribution of diameters (6.23±1.3μm), a uniform hematocrit of 0.45, and dividing by an assumed effective viscosity of 5.84 cP to obtain units of μm^2^. The permeability in the 4 human ROIs was calculated using the same diameter distribution, hematocrit and effective viscosity. The mean EVD of Linninger et al. (2013) was estimated from their histogram of EVDs. Where tortuous capillaries were studied, their mean length or length density was compared to the equivalent values in the human ROIs.

More physiologically-realistic network models have been developed (Safaeian et al., 2011; Su et al., 2012; Linninger et al., 2013; Merrem et al., 2017) to model the cerebral capillaries and to understand the link between structure, blood flow, transit times, and oxygenation in states of hypoperfusion or high metabolic demand (Safaeian et al., 2011; Linninger et al., 2013; Park and Payne, 2016), or the impact of vessel occlusions or radiation damage on capillary function (Su et al., 2012; El-Bouri and Payne, 2015; Merrem et al., 2017). Their main features are summarized next; the difference between metrics reported in these key articles and those in the human ROIs are visualized in Figure 11.

Su et al. (2012) generated two minimum spanning trees which were merged at their end-points, before applying filters to match human capillary length distributions (Cassot et al., 2006). This approach may not replicate the characteristic interconnectedness of the cerebral capillaries; nonetheless, El-Bouri and Payne (2015) found a similar permeability (13% higher) in these networks to that in the synthetic networks developed here (Figure 11).

Other models, like the present work, employed Voronoi diagrams to generate synthetic capillary networks. Safaeian et al. (2011) and Safaeian and David (2013) constructed 2D Voronoi tessellations from uniformly distributed seed points. This was extended to 3D by assigning random angles of deviation, which may produce anisotropic networks. Small sub-networks were stitched together via randomly-placed anastomoses, which could lead to low inter-connectedness. Alternatively, Linninger et al. (2013) generated Voronoi diagrams as the dual of a tetrahedral Delaunay triangulation. After removing excess connections, 86% of vertices were of degree 3, implying that many multiply-connected vertices remained. Finally, Merrem et al. (2017) took a similar approach to the present one with a 3D extension of Lorthois and Cassot (2010), although no pruning of excess vessels was reported, and it was not clear if a three-connected network was obtained.

Until a thorough set of metrics, such as those defined in the present paper, is computed for these different model networks, it is difficult to fully compare the generated structures or validate against anatomical data. Nevertheless, some of these models went further by including additional features to make the networks more physiologically realistic, e.g., a cortical depth-dependent capillary density; capillary tortuosity; links to arborescent arterioles and venules; and a capillary-free zone surrounding larger arteries (Linninger et al., 2013; Merrem et al., 2017). These features could in future be incorporated into the current model.

For example, vessel tortuosity could be added in future studies following Linninger et al. (2013). For flow simulations, its contribution could be assessed by increasing effective vessel lengths by approximately 20%, based on mean tortuous lengths in mice. However the exact spatial location of vessels becomes important when considering EVDs or mass transport (Goldman and Popel, 2000). To give a quantitative idea, EVDs were computed for one mouse ROI with and without tortuosity. The maximum EVD was almost 24% lower with tortuous vessels, suggesting that cerebral capillaries are arranged to avoid large avascular tissue volumes that would be at risk of hypoxia.

Many hemodynamic modeling and simulation studies of brain microvascular structure/function relationships at large scales exploit 3D digital reconstructions of anatomical microvascular networks (Cassot et al., 2006; Mayerich et al., 2008; Tsai et al., 2009; Lorthois et al., 2011a; Gagnon et al., 2015; Peyrounette et al., 2018). However, this does not enable variation of the key structural parameters, e.g., vascular density, in a systematic way. Besides, these models are volume-limited: it is extremely difficult and costly to obtain datasets which resolve all capillaries in very large volumes. This problem could be addressed by generating synthetic capillary networks with *L*_*C*_ tuned to represent distinct brain regions. These could be coupled to anatomical vascular data resolved down to arterioles and venules (Mayerich et al., 2008; Xiong et al., 2017; Di Giovanna et al., 2018) to possibly achieve whole brain flow simulations in mice. Incorporating a parent-daughter diameter correlation and a variation in capillary geometry and topology along flow pathways (Sakadžić et al., 2014), and eventually simulating network remodeling and structural adaptation, or neuro-vascular coupling (Lorthois et al., 2011b; Schmid et al., 2015), would constitute interesting extensions.

The effect of changing microstructural features in pathological scenarios could thus be investigated. The inter-cortical capillary network is highly robust, providing multiple “back-up routes” if a vessel is occluded, whereas the penetrating arterioles are the most “fragile” to occlusions (Nishimura et al., 2007; Hirsch et al., 2012; Shih et al., 2015; Cruz Hernández et al., 2019). The initial study presented here showed that synthetic and mouse capillary networks were similarly robust to single occlusions. Previously, Nishimura et al. (2006) found a mean post- to pre-occlusion red blood cell (RBC) speed ratio of only 7% in the first downstream branches, considering mainly diverging bifurcations. Although RBC speed and blood flow ratios may differ due to post-occlusion vessel dilation, this suggests a more important flow reduction than predicted here (flow ratios of 26–29% in diverging bifurcations). Extrapolating from Nishimura et al. (2010), this could be explained by our focus on purely capillary networks rather than vessels further up the vascular hierarchy (small arterioles or post-arteriole capillaries). Once again, coupling synthetic networks with arterioles and venules will help understand the link between the site of occlusion within the vascular hierarchy and the resulting impact on downstream flows.

Alternately, for larger species for which computational limitations hinder full network simulations, synthetic networks may be used to parameterize continuum models representing the capillary network as a porous medium (Chapman et al., 2008; Hyde et al., 2013; Smith et al., 2014; Peyrounette et al., 2018). Effective properties such as the permeability or mass exchange coefficient could be computed, examining their convergence with domain size and number of networks (El-Bouri and Payne, 2015; Peyrounette et al., 2018); this is not possible for anatomical datasets [here, capillary ROIs were limited to a size of at most (240μm)^3^].

Furthermore, the generation of synthetic vascular networks that recapitulate the architecture, flow, and transport of *in vivo* capillary beds could significantly impact the field of tissue engineering. There has been great interest over the last decade in the generation of large-volume, tissue-engineered constructs. These constructs must contain fluidized vascular networks for transport of nutrients, oxygen, and waste to promote long-term cell survival and function and to mimic physiological and pathological processes (Novosel et al., 2011; Miller, 2014; Kinstlinger and Miller, 2016; Song et al., 2018). Our synthetic networks could be adapted to model different organs (heart, liver, kidney, etc.) according to their specific architecture: the initial Voronoi cell could be modified to introduce variable density or anisotropy. Alternative approaches to controlling the randomness of Voronoi networks (Fritzen et al., 2009) could be investigated. This would greatly facilitate the fabrication of biomimetic vasculature embedded in tissue-engineered constructs via fabrication approaches that rely on 3D image stacks or CAD models to define network geometry (Brandenberg and Lutolf, 2016; Heintz et al., 2016, 2017; Pradhan et al., 2017; Hoon et al., 2018). Additionally, the ability to compare the engineered architecture to a ground truth *in vivo* architecture provides a much needed benchmark to quantify the physiological relevance of engineered microvasculature.

In conclusion, this study has for the first time provided a comprehensive cross-species database of metrics for characterizing the cerebral capillaries. The ability to synthetically replicate cerebral capillary networks, which have equivalent properties according to these metrics, opens a broad range of applications, ranging from systematic computational studies of structure-function relationships in healthy capillary networks to detailed analysis of pathological structural degeneration, or even to the development of templates for fabrication of 3D biomimetic vascular networks embedded in tissue-engineered constructs.

## Author Contributions

SL conceived the study following inspiring discussions with FL, CS, NN, JS, and PB. FL provided human anatomical data. PB, PT, and DK provided mice data. AS, VD, MB, MP, A-EL, and MH-J developed the methods and associated software for synthetic network generation (AS and A-EL), extraction of vascular networks from anatomical data (AS and MH-J), computing blood flow in networks (MP and MB), computing exchange coefficients (VD), computing distance maps (VD) and other metrics (AS). AS generated, post-processed and analyzed all data in the manuscript, including preparing figures and conducting validation studies, with contributions of MB (loop and robustness analysis) and VD (exchange coefficients). AS and SL wrote the manuscript with contributions from VD, YD, MH-J, A-EL, JS, and PB. All authors critically reviewed the manuscript and gave final approval for publication.

### Conflict of Interest Statement

The authors declare that the research was conducted in the absence of any commercial or financial relationships that could be construed as a potential conflict of interest.

## A. Appendix: Definition of Metrics

### A.1. EVD

EVDs were calculated using a Python algorithm to explicitly compute the distance from each 1 μm^3^ voxel in the tissue to the nearest vessel centerline% Amy: ajouter une ref sur la méthode?

The local maxima were defined as voxels with a distance greater than or equal to that of every surrounding voxel in a 26-neighborhood.

A standard box-counting analysis was conducted by dividing the domain into cubes of length *r* and counting the number *N*(*r*) of boxes containing at least one local maxima.

Berntson's procedure was applied to test for linear regimes in the log-log scale plot of *N*(*r*) vs. *r* by searching for at least 4 consecutive points which tested negative for curvilinearity (Berntson and Stoll, 1997; Lorthois and Cassot, 2010).

If a linear regime with slope −*d*_*f*_ is found, the set of local maxima is fractal with fractal dimension *d*_*f*_. If it is homogeneous, there is no linear regime before converging to a slope of −3 at large scales.

To compute the convexity index, Bernston's procedure was again applied to search for a linear regime in the log-log scale histogram of EVDs at small-scales i.e., below *x*_*max*_, the maximum-frequency bin of the histogram. The convexity index was the slope of this linear fit.

### A.2. Flow Solution

Flow simulations were conducted assuming conservation of flux at vertices and a linear pressure drop along vessels, with an effective blood viscosity determined by the *in vivo* viscosity law of (Pries, 2005) with a uniform discharge hematocrit of 0.45. For the human ROIs and synthetic networks modeling human networks, the diameter appearing in the viscosity formulation was divided by 0.86 to account for the difference between red blood cell volumes in humans and rodents Roman et al. (2016).

A pressure drop Δ*P* was imposed on opposing faces of each ROI with a no-flow condition at boundary vertices on the other four faces. El-Bouri and Payne (2015) enforce Δ*P* = 18 mmHg Lorthois et al. (2011a) over a capillary path length *L* = 340μm Sakadžić et al. (2014). Here Δ*P* was scaled for each network to obtain that same pressure gradient Δ*P*/*L* e.g., in the mouse ROIs which have side length 240μm, Δ*P* = 12.7 mmHg.

The resulting linear sparse system of equations was solved via an in-house code Peyrounette et al. (2018).

### A.3. Mass Exchange Coefficient

The two-equation volume averaging method Whitaker (1999) was applied to derive a system of two coupled advection-diffusion equations in terms of the volume-averaged concentrations of a given molecule in vessel and tissue domains, ${\langle C_{v}\rangle }^{v}$ and ${\langle C_{t}\rangle }^{t}$, respectively. These macro-scale equations contain classical advection and diffusion terms, for which we can compute effective diffusion coefficients and effective velocities. Additionally, an exchange term $S=h\left({\langle C_{v}\rangle }^{v}-{\langle C_{t}\rangle }^{t}\right)$, where *h* is the mass exchange coefficient, appears as a source (+*S*) in the tissue-domain equation and a sink (−*S*) in the vessel-domain equation. The effective properties of this upscaled model were obtained by solving a system of Partial Differential Equations (PDEs) on a REV of the domain Cherblanc et al. (2007). These equations were solved by finite element methods, using the library *Feel*++ PrudHomme et al. (2012). The geometry of the domain was taken into account by a fictitious domain method (the level-set method).

Considering the diffusion of a non-reactive, non-metabolic tracer which is highly diffusible through the blood brain barrier, the microscopic a dimensional parameters were reduced to the Péclet number, *Pe*, and the ratio between diffusion coefficients in the tissue and vessel domains, *D*_ratio_ = *D*_*t*_/*D*_*v*_. For given *Pe* and *D*_ratio_, the mass exchange coefficient *h* characterizes the mass transfer properties of the network. The diffusion coefficient in the vessels, *D*_*v*_, was assumed to be 400μm^2^/*s* Clark et al. (1985); Bouwer et al. (1997). Having confirmed that *h* was largely insensitive to *Pe* for a physiological range of velocities, *h* was calculated for *Pe* = 0 and *D*_ratio_ = 0.25. Finally *h* was non-dimensionalized by multiplying by the characteristic time for diffusion for each ROI, i.e., $L_{x}^{2}/D_{v}$, where *L*_*x*_ is the length of the ROI in the *x*-direction.
