# Supplementary material for: Brain Capillary Networks Across Species: A few Simple Organizational Requirements Are Sufficient to Reproduce Both Structure and Function
Source: Front Physiol. 2019 Mar 26;10:233. doi: 10.3389/fphys.2019.00233 (PMC6444172; doi:10.3389/fphys.2019.00233)
Supplement: Supplementary file 1 [file Presentation_1.pdf]

Supplementary material for *Brain capillary networks across species: a few simple organizational requirements are sufficient to reproduce both structure and function (2019) Frontiers in Physiology, doi: 10.3389/fphys.2019.00233.*

by: Amy F. Smith, Vincent Doyeux, Maxime Berg, Myriam Peyrounette, Mohammad Haft-Javaherian, Anne-Edith Larue, John H. Slater, Frédéric Lauwers, Pablo Blinder, Philbert Tsai, David Kleinfeld, Chris B. Schaffer, Nozomi Nishimura, Yohan Davit and Sylvie Lorthois.

S1 Generation of 3D synthetic networks

S1.1 Generation of Voronoi diagram

Each cell of length L_C was divided into $16 \times 16 \times 16$ pixels, and one seed was placed at a random pixel. The 3D Voronoi diagram was extracted using the MATLAB function *voronoin*, which returns the coordinates of vertices and the list of vertices belonging to each polyhedron, from which the network connectivity matrix was constructed.

S1.2 Pruning the network

Very small or narrow polyhedral faces (with area < 75 pixels or with any interior angle $< 30°$) were merged with the neighboring face sharing the longest edge of the current face ie. this edge was deleted. Similarly, neighbouring faces lying almost in a plane (solid angle $< 15°$) were merged. If any triangles with area < 75 pixels remained, the longest edge was removed. Final networks were not very sensitive to the choice of face area or angle criteria.

Vertices were then removed or merged in two stages:

1. *Boundary vertices.* If two boundary vertices $v_{b,1}$ and $v_{b,2}$ were located within some minimum distance D_{min} of each other, $v_{b,1}$ was arbitrarily deleted, but only if it was connected to an interior (i.e. non-boundary) vertex $v_{i,1}$ which had more than three connections. If both boundary vertices were connected to the same interior vertex $v_{i,1}$, which itself only had three connections ($v_{b,1}$ and $v_{b,2}$ plus one other interior vertex $v_{i,2}$), then $v_{b,1}$ and $v_{i,1}$ were deleted, and $v_{b,2}$ was instead connected to $v_{i,2}$. If neither of these cases applied, $v_{b,1}$ was moved a distance D_{min} along the boundary away from $v_{b,2}$ in the direction of the vector between the two vertices.

2. *Interior vertices.* To minimize the computationally heavy task of calculating the distance between all vertices, the domain was divided into sub-cubes of size $L_C \times L_C \times L_C$ (solid lines in 2D in Figure S1a). These cubes were shifted by $0.5L_C$ in each direction yielding an off-set grid, to avoid missing vertex pairs spanning neighboring cubes (dashed lines in Figure S1a). Running through cubes in randomized order, the distance between each pair of vertices within the same cube was calculated. If a pair of vertices (v_1 and v_2) lay $< D_{min}$ from each other, as in Figure S1b, v_1 was arbitrarily deleted and its connections were re-attached to v_2 (Figure S1c), but only if v_2 still had at least three connections. Otherwise, v_1 was moved a distance D_{min} away from v_2 in the direction of the vector between the two vertices.

Final networks were not very sensitive to the choice of D_{min}, and thus its value was fixed at 2.5 pixels (for $L_C = 75\mu$m, $D_{min} = 11.7\mu$m).

Next, excess edges between internal vertices were removed in random order according to the following criteria. An interior vertex could not have more than one connecting edge with a boundary vertex, except if it only had one internal connection, in which case it may be connected to two boundary vertices. If an internal vertex was not connected to any boundary vertices, it should have three connections to internal vertices.

S1.3 Splitting multiply-connected vertices

Vertices with more than 3 connections were treated in random order. For each multiply-connected vertex, the shortest connected edge was kept, as well as the edge with smallest solid angle relative to that

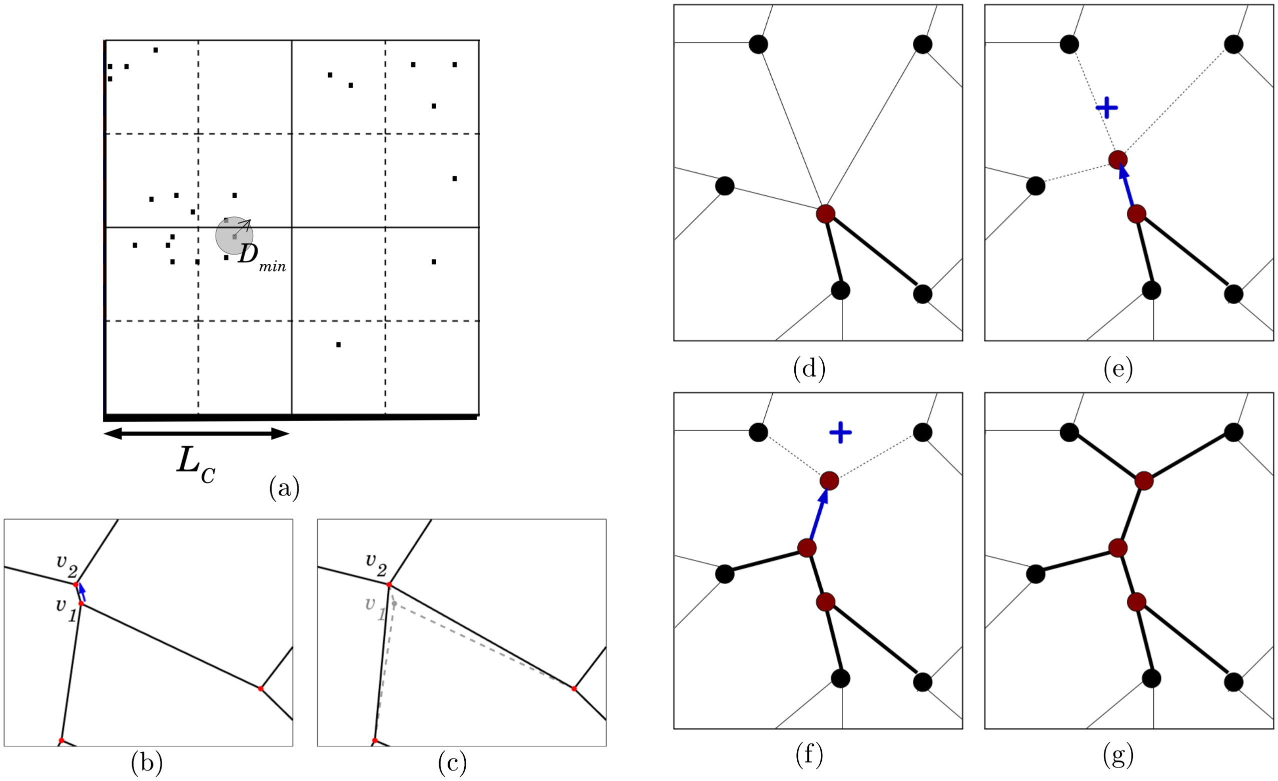

Figure S1: a) Schematic diagram illustrating the division of the domain into a grid of length L_C (solid lines) and an off-set grid shifted by $0.5L_C$ (dashed lines), for efficient detection of vertices (black dots) less than distance D_{min} apart. b) Illustration of two vertices v_1 and v_2 separated by less than D_{min}; c) vertex v_1 and its connections are merged into vertex v_2; d) - g) Sequence of schematic diagrams illustrating the division of a multiply-connected vertex with five connections into three bifurcations. See text for further details.

edge (see e.g. the two edges in bold in Figure S1d). The center of mass of the remaining vertices was calculated (the cross in blue in Figure S1e), and a new vertex was placed on the vector between the initial vertex and the center of mass, at a distance of half the shortest length of the remaining edges, or D_{min}, whichever is smaller (see the blue arrow in Figure S1e). This process was repeated until all interior vertices had only three connections (Figure S1f and g). This step introduced more short vessel lengths, but since there were typically only a small percentage of multiply-connected vertices, this did not have an important effect on overall network properties.

S1.4 Domain size

To avoid any boundary effects associated with the generation of these synthetic networks, for a desired domain size of $L_x \times L_y \times L_z$, a larger network was initially generated in a domain of $2(L_x + 2L_C) \times 2(L_y + 2L_C) \times 2(L_z + 2L_C)$ (with L_i rounded up to the nearest multiple of L_C). At the end of the process, the desired sub-network in a domain of size $L_x \times L_y \times L_z$ was extracted from the center of the large domain.

S2 Results in lattice networks

Results for the two ordered, regular lattice networks introduced in Section 2.2.2 and shown in Figure 1Dare presented. These networks are referred to respectively as the CLN (with 6-connectivity) and the PLN (with 3-connectivity). After a study of the scaling properties of these networks, we present the results of metrics for chosen domain size and scaling.

S2.1 Scaling of lattice networks

We first present an analytical investigation into the scaling of these networks with domain size or with mean length L. Recall that in both lattice networks, the length of the elementary cubes, L_C, is linearly proportional to L ($L_C = L$ in the CLN and $L_C = 3L$ in the PLN).

The Voronoi-like synthetic networks, despite exhibiting greater randomness, also follow a similar semi-ordered structure controlled by the characteristic length L_C. Thus we make the hypothesis that these metrics scale with L_C in the same way, and use this to inform the scaling studies in Section 3.2.

S2.1.1 Morphometrical metrics

The mean vessel length was L for both lattice networks. The length density was $3L/L^3$ for the CLN and $30L/(3L)^3$ for the PLN. Obviously, none of these metrics depended on domain size, in contrast to the loop metrics studied below.

S2.1.2 Topological metrics

The scaling of topological metrics with domain size was derived analytically. Loop metrics computed for lattice networks generated for the range of domain sizes shown in Figure S2 agreed perfectly with the analytical expressions derived in this section.

For both networks, we suppose that i, j and k are the number of unit cells in the x, y, and z directions respectively. Both lattice networks had two modes of loops, $\alpha = 1, 2$, with m_α edges per loop. We then define the mean number of edges per loop, $\overline{N}_{\text{edge/loop}}(i, j, k)$, as the total number of loop edges, divided by the total number of loops, i.e.:

$$\overline{N}_{\text{edge/loop}} = \frac{\sum_{\alpha=1}^{2} m_\alpha N_{loop}^{\alpha}(i,j,k)}{\sum_{\alpha=1}^{2} N_{loop}^{\alpha}(i,j,k)}, \qquad \text{(S1)}$$

where $N_{loop}^{\alpha}(i, j, k)$ are the number of loops corresponding to mode α.

Similarly the mean loop length, L_{loop}, was given by:

$$L_{\text{loop}} = L \times \overline{N}_{\text{edge/loop}},$$

and thus converged with domain size in the same way as $\overline{N}_{\text{edge/loop}}(i, j, k)$, but also scaled with L.

The mean number of loops per edge, $\overline{N}_{\text{loop/edge}}(i, j, k)$, is the total number of loop edges divided by the number of interior (i.e. non-boundary) edges, $N_{\text{edge,int}}(i, j, k)$:

$$\overline{N}_{\text{loop/edge}} = \frac{\sum_{\alpha=1}^{2} m_\alpha N_{loop}^{\alpha}(i,j,k)}{N_{edge,int}(i,j,k)}. \qquad \text{(S2)}$$

These metrics were derived for the specific geometries of the CLNs and PLNs as follows.

Cubic lattice network The CLN had two modes of loops with 4 and 6 edges respectively ($m = \{4, 6\}$). The number of loops with 4 edges, $N_{loop}^{1}(i, j, k)$, followed:

$$N_{loop}^{1}(i,j,k) = i(j-1)(k-1) + (i-1)j(k-1) + (i-1)(j-1)k,$$

for $i, j, k > 0$. Similarly, the number of loops with 6 edges, $N_{loop}^{2}(i, j, k)$, obeyed:

$$\begin{aligned} N_{loop}^{2}(i,j,k) = \; & R\left(i(j-1)(k-2)\right) \\ & + R\left(i(k-1)(j-2)\right) \\ & + R\left(j(i-1)(k-2)\right) \\ & + R\left(j(k-1)(i-2)\right) \\ & + R\left(k(i-1)(j-2)\right) \\ & + R\left(k(j-1)(i-2)\right). \end{aligned}$$

where $R(x)$ is the ramp function, defined as:

$$R(x) = \begin{cases} x, & x \geq 0; \\ 0, & x < 0. \end{cases}$$

Using Equation S1, the mean number of edges per loop for a cube of side length $n = i = j = k$ and $n \geq 2$ was given by:

$$\begin{aligned} \overline{N}_{\text{edge/loop}} &= \frac{4 \times 3n(n-1)^2 + 6 \times 6n(n-1)(n-2)}{3n(n-1)^2 + 6n(n-1)(n-2)} \\ &\to 5^1\!/\!_3 \text{ as } n \to \infty. \end{aligned}$$

The mean number of edges per loop converged to within 5% of the converged value for networks of 4^3 unit cubes (Figure S2a).

In this network, the number of interior (i.e. non-boundary) edges, $N_{\text{edge,int}}(i, j, k)$, was given by:

$$C := R\left((i-1)jk\right) + R\left(i(j-1)k\right) + R\left(ij(k-1)\right). \qquad \text{(S3)}$$

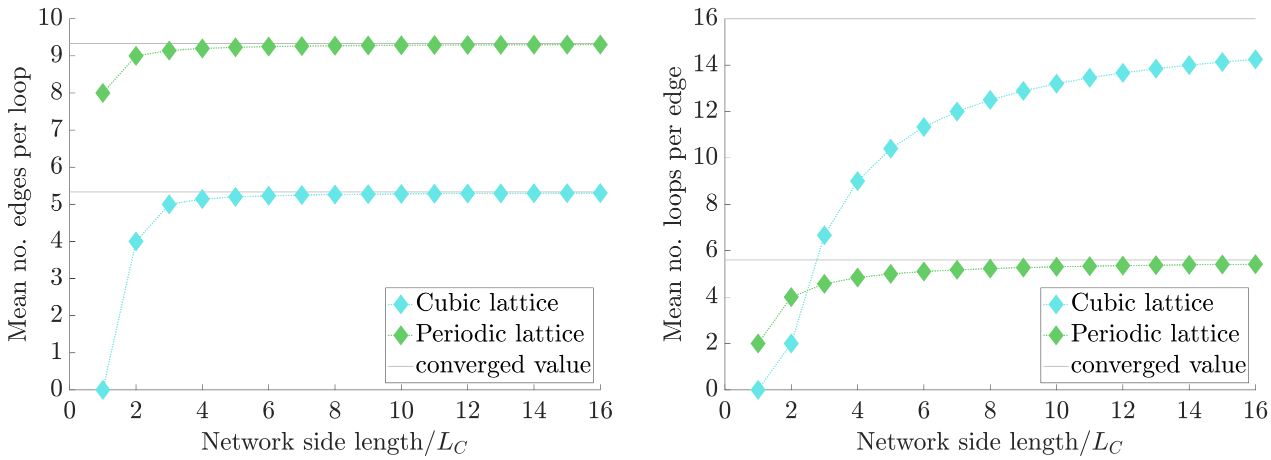

(a) (b)

Figure S2: Scaling of lattice networks with domain size. a) Mean number of edges per loop, $\overline{N}_{\text{edge/loop}}$, and b) mean number of loops per edge, $\overline{N}_{\text{loop/edge}}$, in the CLNs and PLNs as a function of the number of unit cells. Analytical and numerical results agreed exactly. The converged values for each metric and network, computed analytically, are plotted in gray lines.

This quantity C will also be used later. Then, substituting this expression into Equation S2 with $n = i = j = k$ and $n \geq 2$, $\overline{N}_{\text{loop/edge}}$ becomes:

$$\overline{N}_{\text{loop/edge}} = \frac{4 \times 3n(n-1)^2 + 6 \times 6n(n-1)(n-2)}{3n^2(n-1)} \rightarrow 16 \text{ as } n \rightarrow \infty.$$

Convergence was slow: this metric was only converged to within 5% of this value for networks of 36^3 unit cubes or greater (Figure S2b).

Periodic lattice network Next, analytical expressions for the same loop metrics were derived for the PLN, which had two modes of loops with 8 and 10 edges respectively ($m = \{8, 10\}$). The number of loops with 8 edges, $N^1_{loop}(i, j, k)$, followed:

$$N^1_{loop}(i, j, k) = 6ijk,$$

while the number of loops with 10 edges, $N^2_{loop}(i, j, k)$, obeyed:

$$N^2_{loop}(i, j, k) = 4C,$$

where C is defined in Eqn. S3. Using Equation S1, when $n = i = j = k$ and $n \geq 2$, $\overline{N}_{\text{edge/loop}}$ becomes:

$$\overline{N}_{\text{edge/loop}} = \frac{8 \times 6n^3 + 10 \times 12n^2(n-1)}{6n^3 + 12n^2(n-1)} \rightarrow 9^1/_3 \text{ as } n \rightarrow \infty.$$

$\overline{N}_{\text{edge/loop}}$ quickly converged to within 5% of this value for networks of 2^3 unit cubes (Figure S2a).

In this network, $N_{\text{edge,int}}(i, j, k)$ here was given by:

$$N_{\text{edge,int}}(i, j, k) = 24ijk + 2C.$$

Then, using Equation S2, and with $n = i = j = k$ and $n \geq 2$, $\overline{N}_{\text{loop/edge}}$ becomes:

$$\overline{N}_{\text{loop/edge}} = \frac{8 \times 6n^3 + 10 \times 12n^2(n-1)}{24n^3 + 6n^2(n-1)} \rightarrow 5.6 \text{ as } n \rightarrow \infty.$$

$\overline{N}_{\text{loop/edge}}$ converged more quickly for the PLN than the CLN, and was within 5% of its converged value for networks of 11^3 unit cubes or more (Figure S2b).

Thus, in both lattice networks the number of loops per edge was especially sensitive to finite-size effects. Nonetheless, none of the loop metrics except the mean loop length depended on the choice of L (or equivalently L_C), and thus are purely topological metrics.

S2.1.3 Permeability

Cubic lattice network In the CLN, imposing a pressure gradient in the x-direction yields zero flow in vessels orientated parallel to the y- or z-axis: the network is reduced to an array of tubes of length L_x

where L_x is the ROI size in the x-direction. The global flowrate Q_x is:

$$Q_x = \frac{N\pi d^4}{128\mu} \times \frac{\Delta P}{L_x} \tag{S4}$$

where μ is the effective viscosity, d is the diameter (which here is uniform), and ΔP is the pressure drop. N is the number of boundary vessels on the face $x = 0$, expressed in terms of L, the edge length:

$$N = \frac{L_y}{L} \times \frac{L_z}{L}. \tag{S5}$$

From Equation (1), this yields:

$$K_x = \frac{\mu}{\Delta P/L_x} \times \frac{L_y L_z}{A_x L^2} \times \frac{\pi d^4}{128\mu}\frac{\Delta P}{L_x}. \tag{S6}$$

Rearranging and using the definition for the domain area perpendicular to the x-axis, $A_x = L_y L_z$, gives:

$$K_x = \frac{\pi d^4}{128 L^2}. \tag{S7}$$

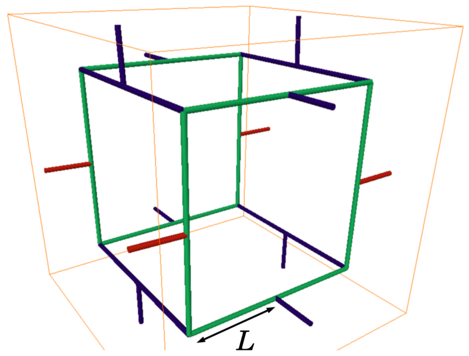

Figure S3: Flow distribution in an elementary motif of the PLN with a pressure gradient from left to right. The magnitude of the flow in the green vessels was half of that in the red vessels, and zero in the dark blue vessels.

Periodic lattice network In the PLN, imposing a pressure gradient in the x-direction with uniform diameters also yields a simple flow distribution (Figure S3 and legend). The global flowrate obeys the same law in Equation S4, but N follows:

$$N = \frac{2L_y}{3L} \times \frac{L_z}{3L}. \tag{S8}$$

In the minimal example shown in Figure S3, $L_y = 3L$ and $L_z = 3L$ giving $N = 2$. Substituting this expression into Equation (1) yields:

$$K_x = \frac{2}{9} \times \frac{\pi d^4}{128 L^2}. \tag{S9}$$

Thus, to obtain the same permeability in both lattice networks, L in the PLN would have to be $\sqrt{2}/3 = 0.47$ times that of the CLN.

Finally, for both lattice networks, the permeability scaled with d^4 and $1/L^2$ (or equivalently $1/L_C^2$)and did not depend on domain size (assuming uniform diameters).

S2.2 Lattice networks scaled to match mouse ROIs

In the CLN, $L = 67\mu$m was chosen to match the length density in mice. A network of $4 \times 4 \times 4$ unit cubes was generated with dimensions $268 \times 268 \times 268\mu\text{m}^3$, to be as close as possible to the size of the mouse ROIs will containing whole unit cubes. In the PLN $L = 41\mu$m; with the unit cube side length being $L_C = 3L$, a network of $2 \times 2 \times 2$ unit cubes was generated with dimension $246 \times 246 \times 246\mu\text{m}^3$. Results in these networks are given in Table 1.

Space-filling metrics In the box-counting analysis, the cut-off lengths for both lattice networks were of a similar order of magnitude to the other networks, but in contrast, both had linear regimes at small scales. The mean EVD was very close to the mouse data for both lattice networks, while the maximum EVD was lowest in the CLN, and highest in the PLN. The mean local maxima of EVDs were 22% and 61% higher in the PLNs and CLNs respectively.

Morphometrical metrics The PLN had a similar mean length to that in mice, while that in the CLN was almost 50% higher. By construction, the length distributions in these networks were drastically different to the mouse and synthetic networks, with only one mode for each network, and hence zero SD. Due to the choice of L as described above, the mean length density in both networks was very close to that in mice. As a result, in the PLN both the edge density and vertex density were close to those in mice, whereas the CLN had a much lower edge and vertex density. Both lattice networks had a much lower density of boundary vertices (36% and 62% respectively).

Topological metrics By construction the PLN had no multiply connected vertices, while all junctions in the CLN had 6 connections. The distri-

bution of loops was very different for the lattice networks, with fewer edges per loop (9 and 5.14 respectively) on average, with values restricted to two modes per network, as described above in Section S2.1.2. The mean loop length was also lower in both lattice networks than in mice.

Flow metrics The mean velocity was 45% higher in both the lattice networks. Similarly, the mean permeability in the PLNs and especially the CLNs was much higher than in mice (almost 30% and 120% higher respectively), and completely isotropic due to their regular construction.

Mass transfer metrics Unsurprisingly, the distribution of capillary transit times for lattice networks were very different to those in the synthetic and mouse ROIs (Figure 8E). The exchange coefficient h was 26.5% and 32.9% higher in the PLNs and CLNs respectively compared mice.

Robustness metrics For the PLN, the histogram of pre- to post-occlusion flow ratios was very different to the distributions for the mice and synthetic networks, due to its highly organized architecture (Figure 8F), and was restricted to 3 modes. The mean flow ratio was approximately 10% lower than in mice. The CLN was not included because none of its vertices were converging bifurcations.

In conclusion, while some simple morphometrical metrics in the lattice networks aligned well with those in mice, their highly organised structure meant that the distribution of metrics was completely different, and topological and functional metrics also differed greatly from the more randomized networks. Thus, such simple networks cannot be used to make accurate predictions of functional properties in the cerebral capillaries.

Supplementary Figures and Tables

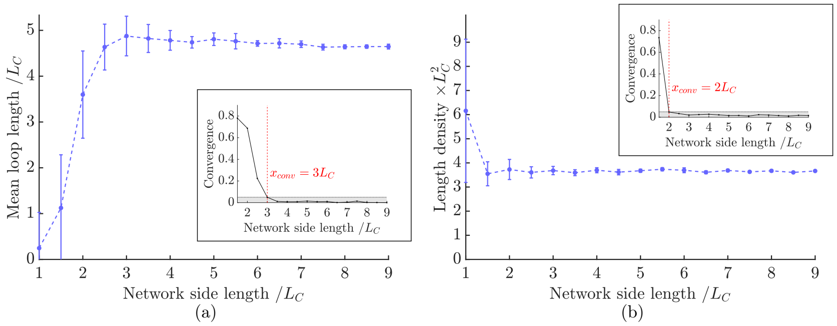

Figure S4: Convergence of metrics with domain size in the synthetic networks: a) mean loop length, b) length density. Metrics were normalized by the appropriate power of L_C. Insets: the convergence of each metric as defined in Equation 2. The converged size x_{conv} is the size from which the convergence was less than 0.05.

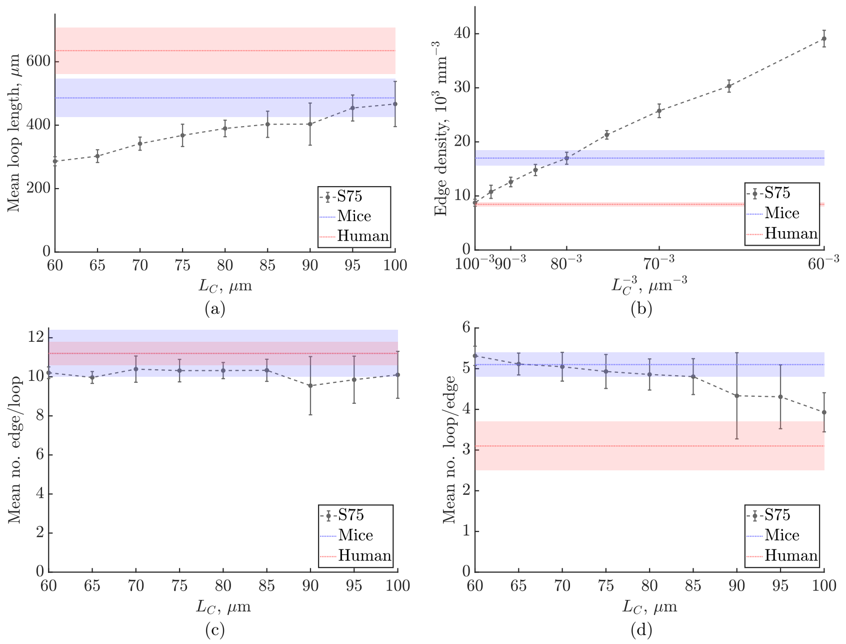

Figure S5: Scaling of metrics with the characteristic length L_C in the synthetic networks: a) mean loop length, b) edge density, c) mean number of edges per loop, d) mean number of loops per edge. Errorbars show mean ± S.D. for the synthetic networks. Shaded bands in blue and red show mean ± S.D. of mouse and human values respectively.

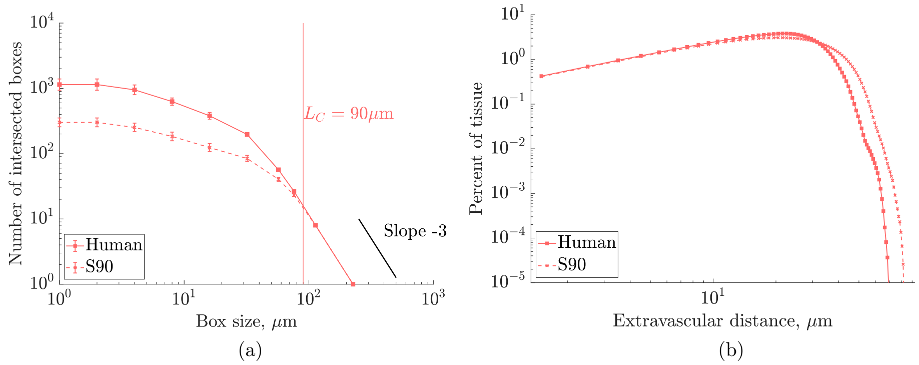

Figure S6: Space-filling results for human and synthetic with $L_C = 90\mu$m networks. a) Box counting results for local maxima of EVDs. For a regular grid of cubic elements of side r, N is the number of square elements containing at least one local maxima. Dotted vertical lines show the value of x_{cut} for each species, above which the slope is -3. b) Histogram of EVDs, collected over all ROIs, on a log-log scale.

Metric	Human	S90
N	4	10
Mean EVD (μm)	21.4 ± 0.6	24.1 ± 1.0
Mean local max EVD (μm)	33.3 ± 1.0	40.2 ± 3.1
Max EVD (μm)	52.9 ± 0.7	63.2 ± 2.7
Convexity index	0.8 ± 0.1	0.7 ± 0.2
Mean length (μm)	60.2 ± 3.1	41.9 ± 1.8
SD length (μm)	41.7 ± 0.8	21.4 ± 2.0
Edge density (10^3 mm^{-3})	8.4 ± 0.4	11.9 ± 0.9
Length density (mm^{-2})	461 ± 19	442 ± 28
Vertex density (10^3 mm^{-3})	3.9 ± 0.2	6.3 ± 0.5
Boundary vertex density (mm^{-2})	207 ± 8	186 ± 16
% multiply-connected vertices	2.9 ± 1.7	2.6 ± 1.6
Mean no. edge/loop	11.2 ± 0.6	10.2 ± 0.6
Mean loop length (μm)	635 ± 73	429 ± 34
Mean no. loop/edge	3.1 ± 0.6	4.4 ± 0.5
Mean velocity (μm/s)	148 ± 34	202 ± 26
SD velocity (μm/s)	223 ± 34	228 ± 18
Permeability ($10^{-3}\mu$m^2)	0.62 ± 0.21	0.86 ± 0.13
Median transit time (s)	0.19 ± 0.06	0.14 ± 0.02
Exchange coefficient h	27.7 ± 3.71	15.7 ± 1.12
Post-occlusion flow ratio (converging)	0.77 ± 0.03	0.76 ± 0.01
Post-occlusion flow ratio (diverging; branch A)	0.28 ± 0.05	0.28 ± 0.04

Table S1: The geometrical, topological and functional metrics calculated here, for human and synthetic with $L_C = 90\mu$m ('S90') networks. Results are presented as mean ± S.D. over the N ROIs studied for each network type. Permeabilities, velocities and transit times were calculated with uniform diameters of 5μm.

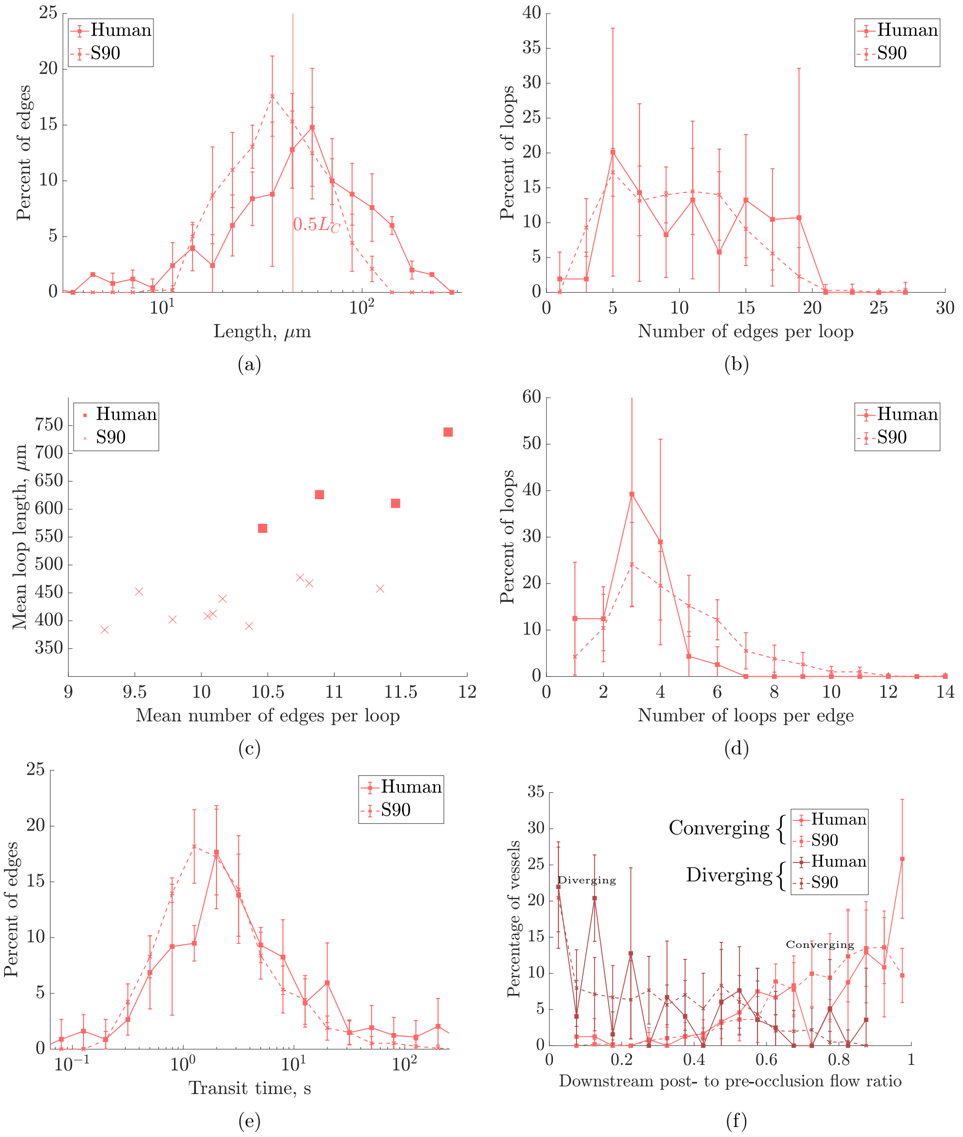

Figure S7: Results for human ROIs and synthetic networks ('S90') with $L_C = 90\mu$m and domain size $264 \times 264 \times 207\mu\text{m}^3$. a) Histogram of lengths on a log-scale. Frequencies collected over all ROIs for each network type. b) Histogram of number of edges per loop. c) Mean loop length, μm, vs. mean number of edges per loop, for each ROI. d) Histogram of number of loops per edge. e) Histogram of capillary transit times, on a log-scale. f) Histograms of post- to pre-occlusion absolute flow ratios in vessels one branch downstream from the occlusion, where the vertex downstream of the occlusion has 3-connectivity, and divided into converging and diverging bifurcations. In the diverging case, flow ratios are plotted for the outflow branch without change in flow direction post-occlusion.

Metric	Unit	n	α	β	R^2
Length density	mm^{-2}	-2	3.70×10^6	0.61	0.996
Mean EVD	μm	1	0.26	0.33	0.991
Permeability	μm^2	-2	9.43	-2.05×10^{-4}	0.995
Mean loop length	μm	1	4.45	23.23	0.971
Mean length	μm	1	0.41	4.27	0.991
Edge density	mm^{-3}	-3	8.20×10^9	1.21×10^3	0.998

Table S2: In the synthetic networks, results of a standard linear regression on a range of metrics as a function of L_C to the appropriate power, i.e. following the expression: $\alpha L_C^n + \beta$, where n is the power appropriate for each metric. The coefficient of determination, R^2, was very good for all fits.
